# Wearable monitoring of pre-shot heart rate in youth golfers: a potential mental-load correlate in ecological putting practice

**DOI:** 10.3389/fpsyg.2026.1939721

**Published:** 2026-10-28

**Authors:** Jasper Gielen, Katrien Buysse, Elise Heirman, Romain Meeusen, Jean-Marie Aerts

**Affiliations:** 1 Research Group Measure, Model and Manage Bioresponses (M3-BIORES), KU Leuven, Biosystems Department, Leuven, Belgium; 2 Human Physiology and Sports Physiotherapy Research Group (MFYS), Vrije Universiteit Brussel, Brussels, Belgium; 3 Department of Sports, Recreation, Exercise and Sciences [SRES], Faculty of Community and Health Sciences, University of the Western Cape, Cape Town, South Africa

**Keywords:** coach–athlete feedback, ecological validity, golf putting, heart rate, mental load, mixed-effects models, physical activity, wearables

## Abstract

**Introduction:**

Information and communication technologies, including wearable sensors, offer accessible and ecological ways to support psychological processes underpinning technical practice in sport, such as the regulation of attention, arousal and mental load. The aim of this exploratory study is to examine whether heart rate (HR) and physical activity (PA), recorded with a chest-worn wearable, can serve as a potential, indirect correlate of the mental load experienced during golf putting training. Two research questions were of interest: (i) Do pre-shot HR and PA differ between made and missed putts? (ii) Do pre-shot HR and PA differ following a made compared with a missed putt?

**Methods:**

Seven youth golfing athletes (13–16 years old; handicap (hcp) index < 12) performed a putting training exercise in which the goal was to score five consecutive putts from 3-metre distance to the hole. HR and PA were recorded continuously and matched with putting performance using video footage. Pre-shot HR and PA were taken as the mean over the 5 s before ball contact. Because the putts are nested within sessions and athletes, the analyses combine within-athlete non-parametric tests, corrected for multiple comparisons and reported with effect sizes, with linear mixed-effects models treating athlete and session as random effects.

**Results:**

Pre-shot HR and PA do not differ significantly between made and missed putts (mixed-model estimate for HR: +0.09 bpm, 95% CI [−0.46, 0.64]; *p* = 0.76). However, pre-shot HR is higher following a made putt and lower following a missed one (+5.08 bpm, 95% CI [4.54, 5.62]; *p* < 0.001), an association that persists after adjusting for the pre-shot HR of the preceding putt. PA shows the opposite trend.

**Discussion:**

Because no subjective ratings of mental load, arousal or affect were collected, mental load remains one plausible interpretation of this pattern rather than an established one. Pending validation against such measures, the approach could offer coaches and athletes an exploratory starting point for conversations about how training situations are experienced, and illustrates how continuous monitoring of HR may provide a potential indirect correlate of mental load in youth golfers.

## 1 Introduction

Golf is widely regarded as a mentally demanding sport, in which psychological factors such as anxiety, attentional control and the ability to perform under pressure can materially affect performance (Hill et al., 2010; Cotterill, 2010). This view is supported not only by the accounts of professional athletes and coaches, but also by a substantial body of sport-psychology research on stress and performance in golf. In turn, researchers have tested this by studying the cognitive loads experienced by elite, amateur and novice golfers. As golf not only requires great physical skill and motor function, but also great cognitive function (e.g., awareness, concentration; Clark et al., 2005), different methods for quantifying the latter have been developed.

For instance, electroencephalography (EEG) measurements show how different regions in the brain attribute to the attentional focus associated with golf putting performance (Crews and Landers, 1993). Alternatively, gaze behaviours have been assessed to determine focus of elite and non-elite athletes (Singer, 2000). A method that has proven to consistently discriminate elite golfers from the rest is the quiet eye (QE; Vickers, 2012). The QE is the final fixation or tracking gaze located on a specific target for more than 100 ms (Vickers, 1996). The onset of the QE in elite golfers occurs earlier and the duration is longer compared to non-elites (Vickers, 2012). Moreover, MRI studies of subjects (including golfers) match the known patterns in brain activity with the observed QE behaviour (Vickers, 2012).

While the earlier mentioned methods are essential to gain fundamental insights into the complex processes underlying golfing, the applicability in (daily) routines of the athletes is far from straightforward. As an alternative, heart rate (HR) has also been associated with mental load such as the preparation and initiation of target-aiming tasks (e.g., golfing; Boutcher and Zinsser, 1990; Lacey, 1967). Moreover, the dynamic behaviour of HR measurements has been linked to golf-specific performance levels (Boutcher and Zinsser, 1990; Henderson et al., 2024; Neumann and Thomas, 2009, 2011).

Although wearables equipped with continuous HR monitoring are widespread and even used in training by professional and amateur golfers, the technology is currently not leveraged for mental load monitoring. Given its potential, applying wearables in this specific context will be our primary focus.

More broadly, this study is positioned within the growing use of information and communication technologies (ICT) to support athlete development. Beyond refining technique and tactics, digital tools such as video feedback, virtual reality and wearable sensors increasingly make it possible to target the psychological processes that underpin skilled performance, including attention, arousal regulation and self-regulation. Wearables are a particularly accessible and ecological example: by rendering otherwise invisible physiological states available to athletes and coaches, they could in principle act as a feedback tool that supports awareness and self-regulation of mental load during technical practice. We stress that in the present study no feedback was delivered to athletes or coaches, and no self-regulation outcome was assessed. What is presented here is whether the physiological signal such an application would have to rely on is measurable, and interpretable, in an ordinary training session. Self-regulatory skills are known to distinguish more- from less-successful youth athletes (Jonker et al., 2010), which makes the developmental context of this study (i.e., skilled but still-developing youth golfers) especially pertinent.

Within the context of studying cognitive functioning and mental loads during golfing, research often focusses on putting. From a practical point of view, the limited amount of physical activity during putting compared to a full swing, increases the accuracy and reliability of the data collection. Apart from practical considerations, putting is also simply one of the most important strokes in golf (Gryc et al., 2021). Professionals play 43% of the strokes with the putter in a single round of golf (Pelz and Frank, 2000). Additionally, research has focused on golf putting for assessing the use of pre-performance routines to mentally and physically prepare themselves for the execution of an (athletic) skill (Bell et al., 2010). For instance, a solid pre-shot routine is deemed necessary for success in golf (Clark et al., 2005), and a recent meta-analysis shows the benefits of pre-performance routines in practice, regardless of the type of routine that is applied (Rupprecht et al., 2021).

This pre-shot phase has been examined extensively. Prior work has compared pre-shot routines between amateurs and professionals and between more and less successful competitors, and has manipulated pressure both in the laboratory and in the field, drawing on video analysis, electrocardiography, eye-tracking and heart-rate monitoring (Bell et al., 2010; Cotterill, 2010; Mesagno and Mullane-Grant, 2010; Cooke et al., 2014; Vine et al., 2011). A recurring theme is the capacity to deliver so-called ‘clutch’ performance (i.e., successful execution under acute pressure), which has been proposed to distinguish more-skilled from less-skilled performers and is particularly pertinent to short putts that are expected to be holed (Otten, 2009; Swann et al., 2017; Hill et al., 2010). Against this well-developed background, the present study does not set out to uncover new mechanisms of the pre-shot routine. Its contribution is instead methodological and applied: it uses an unobtrusive, low-cost wearable to monitor HR and PA continuously across a large number of putts during a regular training exercise, and examines how these signals change as a function of the preceding outcome and the running score.

For the above-mentioned reasons, our focus on leveraging wearables is specifically targeted on applications for golf putting. HR and accelerometer-based physical activity (PA) measurements are particularly relevant to mental load monitoring because they reflect underlying autonomic and motor system responses to cognitive demands. HR dynamics, including increases or decreases during task preparation, have been linked to attentional focus, arousal, and task difficulty (Cooke et al., 2014; Neumann and Thomas, 2011). Similarly, PA levels, can provide complementary insights into motor readiness or compensatory behaviours associated with cognitive strain (Boutcher and Zinsser, 1990). These metrics are non-invasive, easily monitored in real-world settings, and capable of capturing physiological changes that accompany mental effort during goal-directed tasks such as golf putting.

Hence, the aim of this study is to explore whether HR [together with physical activity (PA)] measured with wearable technology can serve as a potential, indirect correlate of the mental load experienced during golf putting, while recognising that these signals are shaped by many factors besides mental load and that a cardiac response alone cannot establish which of them is operating. Here, mental load is defined as increases in the cognitive demands of a task (i.e., putting), which entails a rise in the recruitment of processing resources (Shuggi et al., 2017). More specifically, HR and PA are studied in field-conditions during putting practice sessions that are part of the study participants’ regular training schedule. By bringing this research out to real-world golf training situation, we aim to bridge the gap between laboratory studies on mental load monitoring and the actual training environment of athletes and coaches. Moreover, two particular research questions were of interest: (i) Do HR and PA metrics during the pre-shot phase differ between made and missed putts? (ii) Are the HR and PA metrics during the routine of a putting attempt associated with the outcome of the preceding shot (i.e., made or missed)?

Because the terms used in this literature are closely related but not interchangeable, we define them explicitly here and use them consistently for the remainder of the manuscript. Mental load, which we use synonymously with cognitive load, refers to the demand a task places on the limited processing resources of the performer and to the effort recruited to meet that demand (Sweller, 1988; Paas et al., 2003; Shuggi et al., 2017). Arousal refers to the intensity dimension of physiological and affective activation, independently of whether the accompanying experience is pleasant or unpleasant (Russell and Barrett, 1999). Stress and anxiety refer to the appraisal of a situation as threatening and to the negatively valenced cognitive and somatic responses that follow (Martens et al., 1990; Eysenck et al., 2007). Pressure, finally, is a property of the situation rather than of the performer: it denotes circumstances in which the incentive for performing well is unusually high (Baumeister, 1984; Carr, 2015). These constructs are correlated, and every one of them can raise heart rate, which is precisely why a cardiac response on its own cannot identify which of them is operating.

## 2 Materials and methods

### 2.1 Participants

Seven youth golfing athletes participated in this study (six males; one female). Four athletes were 16 years old and had a hcp index of less than or equal to 4. Three athletes were 13 years old and had a hcp index of less than or equal to 12. The hcp index or ‘handicap’ in golf indicates the level of skill. An index of zero means that the player is able to complete a round of 18 holes consistently without requiring extra strokes beyond the recommended number by the course designers (typically 70–72). A handicap index below 12 is considered to be a very good level (Vickers, 1992). We note that a handicap index in this range denotes a skilled, competitive golfer rather than an objectively elite or ‘high-level’ performer; the participants are therefore described here as skilled youth golfers of varying standard. All athletes were enrolled in a high school programme that specialises in the development of talented youth golfers.

The sample comprised the seven youth golfers who were enrolled in the collaborating talent-development programme and available across the data-collection period; it is thus a convenience sample rather than an a-priori powered one, and all analyses are performed within individuals to accommodate the resulting variability in standard and in the number of putts. The group was predominantly male (six males, one female), reflecting the composition of the programme and precluding any inference about sex differences. The researchers were not the athletes’ personal coaches; data were collected during regular training sessions with the cooperation of the coaching staff, and the researchers’ role was confined to instrumentation and observation. The heterogeneity in playing standard is a limitation for between-athlete comparison, but is representative of a real training group and supports the ecological relevance of the observations.

### 2.2 Study design

The test protocol was designed as a training exercise for golf putting. Ten different positions were marked at a distance of 3 m from the hole on the putting green. The positions were equally spaced around the hole, forming a circle. The goal of the exercise was to score five consecutive putts. With each attempt, regardless of making or missing the putt, the athlete moves to a different marker and takes the shot from the next position. This ensures that no two consecutive putts were performed from the same spot on the green. Moreover, the setup ensures that the walk between attempts was short and of comparable duration irrespective of the outcome. After a made putt the athlete retrieved the ball from the hole; after a miss the ball was retrieved from where it came to rest. Walking distance and walking pace between attempts were nevertheless not measured. The 3-metre distance to the hole is commonly used in golf-putting research (e.g., Gryc et al., 2021; Vickers, 1992; Vine et al., 2011). Figure 1 visualises the set up on the putting green. The exercise was self-paced, meaning that the golfers were free to take as much time as they want to perform the shots and they only finish when scoring five consecutive times. We emphasise that this protocol is a structured training exercise embedded in the athletes’ regular practice, and not a competitive round or tournament. Although the ‘five-in-a-row’ requirement is intended to create self-imposed pressure, the outcome carried no competitive consequence such as a stroke on a scorecard, and the results should be read with this distinction in mind.

**Figure 1:**
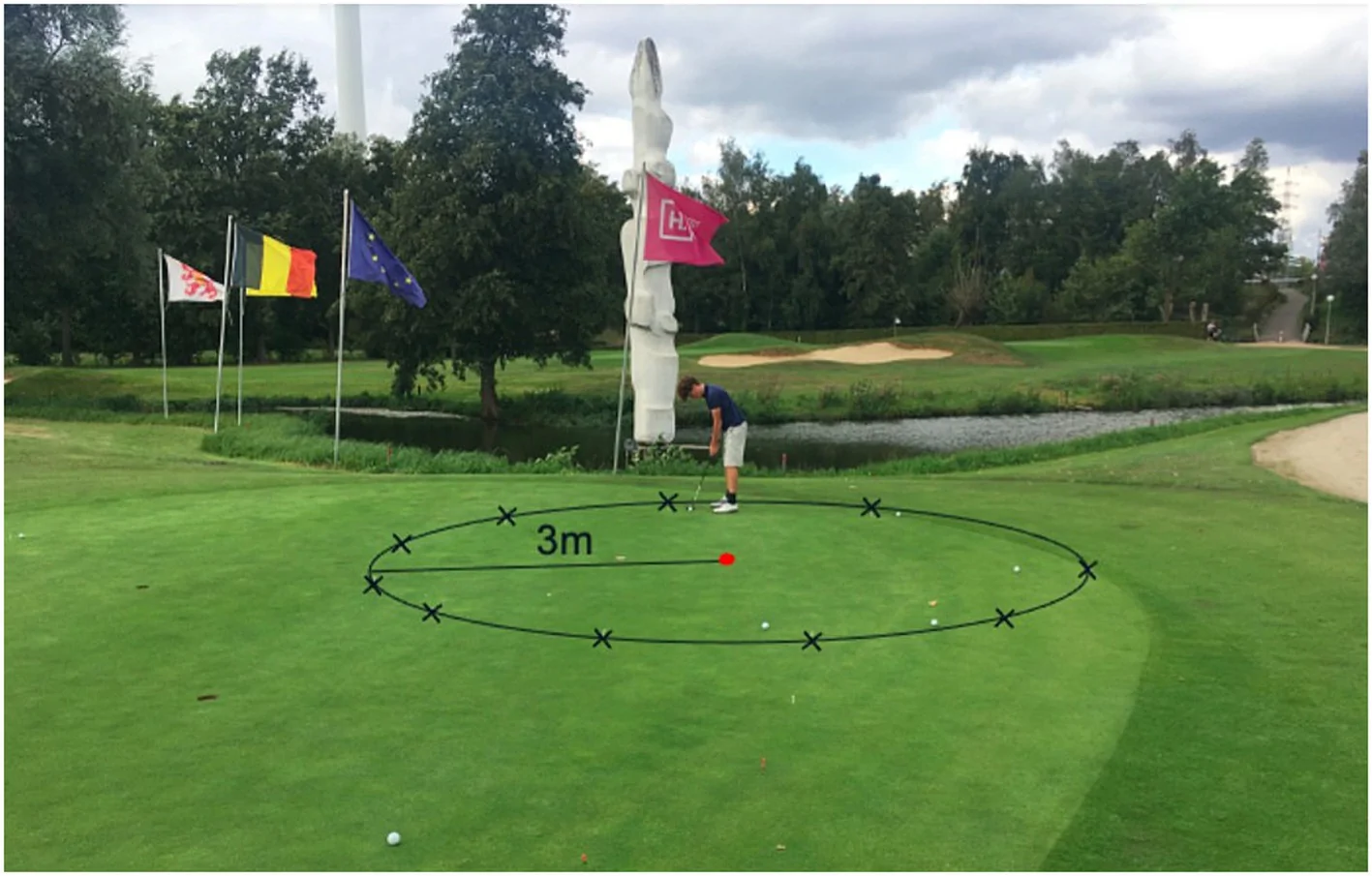
Set up for the putting exercise on the green. The X’s mark the position for the ball and are indicated on the grass with a small marker (e.g., golf tee).

Throughout the sessions, a Zephyr Bioharness 3.0 (Medtronic, Boulder, CO) was worn around the chest to continuously measure heart rate (HR) and physical activity (PA) at 1 Hz. Studies on the validity of the Zephyr Bioharness system yielded good to excellent accuracy for the HR measurements (Nazari et al., 2018). The device provides an automatic calculation of the HR signal, and a confidence measure for the quality of the underlying ECG recordings. The device reports this confidence measure for every 1 Hz sample. In line with the device guidelines, samples with a confidence value at or below 30% were replaced by missing values before any averaging was performed. Across the recordings retained for analysis, the sample-wise confidence exceeded 80% for the whole of every session. Four complete recordings that showed poor ECG confidence throughout were excluded at the data-preparation stage and are therefore not among the 29 sessions reported here. The PA was determined as the root-mean-squared value of three orthogonal accelerometers. The resulting measure for PA is expressed in gravitational units (1 GU = 9.81 m/s^2^). Although the primary focus of the study lies on analysing the HR data, the accelerometer data provides crucial information to put the cardiovascular responses into context. Upper body accelerometry is considered to provide a robust indication of PA levels during putting. Furthermore, video footage was used to label the putts as ‘made’ or ‘missed’ and add timestamps to the events.

The study design was approved by the Social and Societal Ethics Committee (SMEC) of KU Leuven. Data were collected in two consecutive measurement campaigns, each covered by its own approval: cases G-2020-2451-R2(MAR) and G-2022-5794-R2(MAR). The exercise, the instrumentation and the labelling procedure were identical in both campaigns, which is why the data are pooled here. Written informed consent was obtained from the parents or legal guardians of every participant, and written assent was obtained from each youth athlete personally before participation. The data processing and statistical testing described hereafter were performed using MATLAB R2021a (The MathWorks Inc., Natick, MA).

### 2.3 Data processing

The collected data comprised time-series for HR and PA with timestamps and labels of the performed putts. For each putt, the pre-shot levels of HR and PA were determined by taking the mean value in a window of 5 s prior to hitting the ball. The pre-shot data were chosen to reflect the preparation for each putt. The duration of 5 s reflects the definition of the ‘pre-shot phase’ by Crews and Boutcher (1986). Pre-shot windows of 10 and 15 s were analysed alongside the 5-s window, using the same models and corrections (Supplementary Table S1). In addition to these absolute values of pre-shot data, the change in pre-shot HR and PA between consecutive putts is also determined. These metrics allow to identify how the pre-shot values may change after making or missing a putt. Furthermore, the phasic decrease and deceleration rate of the pre-shot HR is also determined. Both metrics have been linked to task difficulty and the mental load of golf putting in literature (Henderson et al., 2024). The phasic HR decrease is computed as the difference between the highest and the lowest HR values in a 10-s window prior to contact with the ball. The rate of HR deceleration was computed as the HR decrease divided by the time between the two heart rate samples. These calculations are in line with the methods used in related literature (Cooke et al., 2014; Neumann and Thomas, 2009, 2011). In addition to these pre-shot physical and physiological data, the outcome of each putt was labelled as ‘Made’ or ‘Missed’. These binary labels are also used to determine the score over the course of the putting exercise, which reflects the number of consecutive putts for any given attempt. The score can vary from 0 (last attempt missed) to 5 (holed five consecutive putts; exercise is completed).

### 2.4 Statistical analyses

The analyses proceed in two complementary steps. The first step follows a within-athlete, non-parametric approach. This is deliberate: the seven athletes differ substantially in resting heart rate, in playing standard and in the number of putts contributed, so pooling raw values across athletes would confound between- and within-athlete variation. To identify differences associated with making or missing putts, Wilcoxon rank sum tests for unpaired, non-normally distributed data were performed within each athlete. To test whether changes in HR and PA between consecutive putts differ from a zero median, Wilcoxon signed rank tests were used. Kruskal-Wallis tests were used to compare data distributions across more than two groups, followed by Dunn’s post-hoc test for all pairwise comparisons whenever the omnibus test was significant (Dunn, 1964). The analyses by score are exploratory: they were not planned before data collection, and the Kruskal-Wallis test is an omnibus test that is indifferent to the ordering of the score levels, so no formal test of a monotonic trend across scores was performed. They are reported to generate hypotheses rather than to test them. The normality of the data was tested before selecting these non-parametric tests.

Every test is accompanied by reporting of the corresponding effect size. For the two-sample comparisons we report the rank-biserial correlation, which is the difference between the proportion of cross-group pairs favouring each label and ranges from −1 to +1 (Kerby, 2014); for the one-sample comparisons we report the matched-pairs rank-biserial correlation; and for the Kruskal-Wallis tests we report epsilon-squared. Because effect sizes computed on putts that are clustered within sessions have optimistically narrow conventional confidence intervals, 95% confidence intervals were obtained by a cluster bootstrap that resamples whole sessions (2000 replicates) rather than individual putts.

The analyses comprise a family of tests per metric and per research question, one for each athlete. All *p*-values are therefore reported both unadjusted and after correction for multiple comparisons. Correction was applied within each pre-declared family of seven athlete-wise tests, using both the Holm-Bonferroni procedure and the Benjamini-Hochberg false discovery rate procedure (Holm, 1979; Benjamini and Hochberg, 1995). Where the two procedures lead to different conclusions, this is stated explicitly. The significance level was set to 0.05 for every test.

The second step addresses the fact that all putts are not independent observations. They are nested within 29 sessions, which are in turn nested within seven athletes, and consecutive putts within a session are physiologically autocorrelated: the median within-session lag-1 autocorrelation of the pre-shot HR is 0.40 (interquartile range 0.31 to 0.52). Treating individual putts as independent would inflate the apparent precision of any pooled estimate (Hurlbert, 1984; Lazic, 2010; Aarts et al., 2014). We therefore fitted linear mixed-effects models (MATLAB fitlme, restricted maximum likelihood) of the form metric ~ 1 + outcome + (1 | athlete) + (1 | athlete:session), in which the putt outcome enters as a fixed effect and random intercepts absorb the between-athlete and between-session variation.

These mixed-effects models are the confirmatory analysis of this study; the athlete-wise tests are presented as a description of how consistent the pattern is across individuals, not as independent evidence. Estimates are reported in the original units (bpm for HR, gravitational units for PA) with 95% confidence intervals, so that the practical magnitude of an effect can be judged separately from its statistical significance. Because a random-intercept model still assumes conditionally independent residuals, each model was refitted with the pre-shot value of the preceding putt as an additional, mean-centred covariate; this lag-1 adjustment absorbs the serial dependence directly and provides a stringent check on the reported effects. Finally, the intraclass correlation coefficients derived from the variance components describe the variance structure of the data, that is, how much of the total variance is attributable to differences between athletes and to differences between sessions of the same athlete (Koo and Li, 2016). Together with the within-session coefficient of variation and the session-wise results, they are reported as indices of within- and between-session variability. They are not a comprehensive reliability assessment: no test–retest, agreement or measurement-error analysis was performed, and the design does not support one. We also stress that modelling the clustering corrects the precision of the pooled estimates but does not overcome the limited population-level sample. The 3,691 putts increase the precision with which each athlete’s response is estimated; they do not increase the number of individuals from which a population-level inference could be drawn, and seven athletes therefore remain the effective unit of generalisation.

## 3 Results

All athletes performed the putting exercise multiple times and on separate occasions. The number of repetitions depended on their availability (i.e., training schedule, injury, illness, etc.). Table 1 presents how many times each athlete performed the putting drill. Table 1 also contains information about the total number of putts during each session as well as which portion of those were successful. In total, 29 individual measurements are included in the analyses. In total, 3,691 putts were performed of which 1,218 were successful (= 33.0%). Because putts are nested within sessions and sessions within athletes, the results below are reported at three levels: pooled mixed-effects estimates that treat athlete and session as random effects and constitute the confirmatory analysis, athlete-wise tests with effect sizes and multiplicity-corrected *p*-values that describe how consistent the pattern is across individuals, and session-wise tests that describe how consistent it is over time.

**Table 1:** Overview of the number of sessions and putts performed.

Session	Athlete 1 16 y/o (M)	Athlete 2 16 y/o (M)	Athlete 3 16 y/o (F)	Athlete 4 16 y/o (M)	Athlete 5 13 y/o (M)	Athlete 6 13 y/o (M)	Athlete 7 13 y/o (M)
Session 1	38 (58%)	135 (44%)	376 (30%)	79 (37%)	44 (32%)	198 (36%)	264 (35%)
Session 2	110 (37%)	138 (30%)	117 (23%)	27 (52%)	81 (37%)	148 (36%)	139 (40%)
Session 3	176 (39%)		61 (18%)	20 (25%)	314 (28%)	44 (55%)	206 (43%)
Session 4	26 (50%)				299 (13%)	23 (61%)	70 (34%)
Session 5	21 (24%)				40 (48%)	92 (36%)	308 (29%)
Session 6					97 (33%)		
Total	371 (40%)	273 (37%)	554 (27%)	126 (38%)	875 (25%)	505 (39%)	987 (36%)

In brackets are the success rates. Athletes’ age and sex (M/F) are indicated for each individual.

The first analyses are related to the research question whether HR and PA are predictive for the outcome of a given putt. The pre-shot HR and PA data linked to making or missing putts are presented in Figure 2 for each individual athlete. The distribution of the average pre-shot HR and pre-shot PA for both labels are shown in the upper panels. In addition, the phasic HR decrease and deceleration rate are displayed in the lower panels. Wilcoxon rank sum tests indicate no significant differences between the labels of ‘Missed’ and ‘Made’ putts for the three different pre-shot HR metrics; the corresponding rank-biserial correlations are small and their confidence intervals include zero for every athlete (|r| ≤ 0.10 for the pre-shot HR; Supplementary Table S2). Regarding the pre-shot PA, one athlete (A7) recorded higher activity levels before successful putts (*p* = 0.041, *r* = 0.08, 95% CI [−0.01, 0.16]), but this difference does not survive correction for the seven athlete-wise tests (Holm- and FDR-adjusted *p* = 0.29) and is therefore best regarded as a chance finding. The mixed-effects models reach the same conclusion with all athletes pooled. The outcome of the upcoming putt is associated with a difference in pre-shot HR of +0.09 bpm (95% CI [−0.46, 0.64], *p* = 0.76) and in pre-shot PA of +0.004 g (95% CI [−0.001, 0.008], *p* = 0.11); adjusting for the pre-shot HR of the preceding putt leaves this unchanged (+0.09 bpm, 95% CI [−0.39, 0.57], *p* = 0.72). It is worth noting that the confidence interval for HR excludes any effect larger than roughly 0.6 bpm, which is well below what could plausibly be of practical use to a coach. The absence of a predictive relation is therefore an informative null result rather than a matter of insufficient sensitivity.

**Figure 2:**
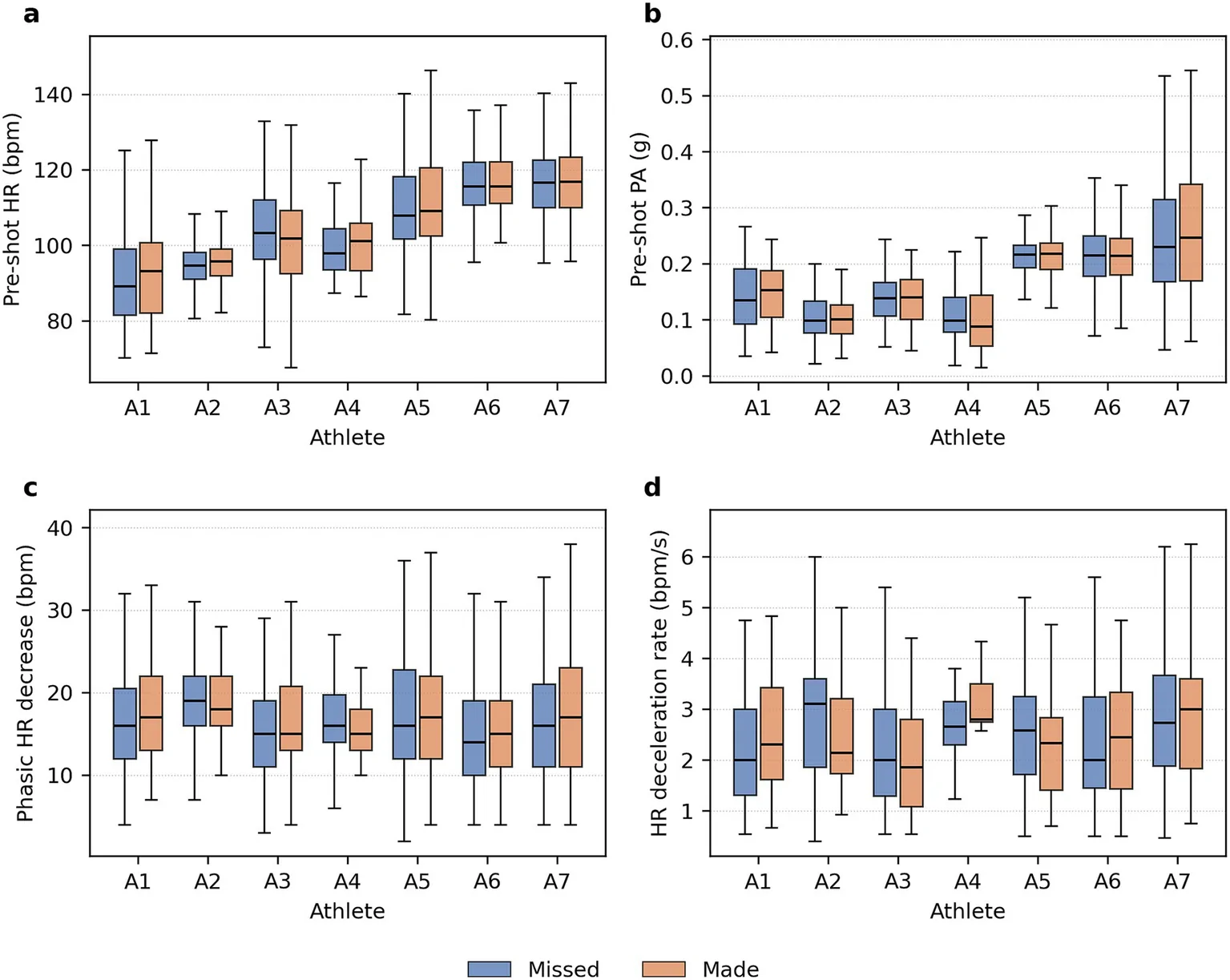
Distribution of the pre-shot HR (panel **a**), accelerometer-based PA levels (panel **b**), phasic HR decrease (panel **c**), and phasic HR deceleration rate (panel **d**) corresponding to whether the preparation results in a ‘Missed’ or a ‘Made’ putt for each athlete (‘A1’–‘A7’). The distributions are shown as the median value (central line) and the interquartile range, with whiskers extending to 1.5 times the interquartile range. No significant differences are observed (Supplementary Table S2).

Next, the pre-shot HR and PA data are matched with the outcome labels of the preceding putt. Figures 3a,b (upper left and right panel) shows that for this case, there are differences between the distribution of absolute values of the pre-shot HR and PA. For six of the seven athletes, the pre-shot HR is higher when the prior putt was made than when it was missed, and this difference remains significant after correction for multiple comparisons (FDR-adjusted *p* ≤ 0.002); the rank-biserial correlations range from *r* = 0.16 (95% CI [0.03, 0.34]) for A6 to *r* = 0.58 (95% CI [0.52, 0.79]) for A4. The single exception is A3, for whom the median pre-shot HR is 1.9 bpm lower after a made putt and the difference is not significant (*p* = 0.40, *r* = −0.05). In other words, after scoring a putt, the pre-shot HR increased for attempting the next shot in six of the seven athletes. When comparing the same situation for the pre-shot data of PA, the activity levels are constant or decreased. For three athletes, the observed decrease in the pre-shot activity is statistically significant after correction (A3, A4 and A7; FDR-adjusted *p* < 0.001), with the largest effect for A4 (*r* = −0.53, 95% CI [−0.73, −0.25]; Supplementary Table S3).

**Figure 3:**
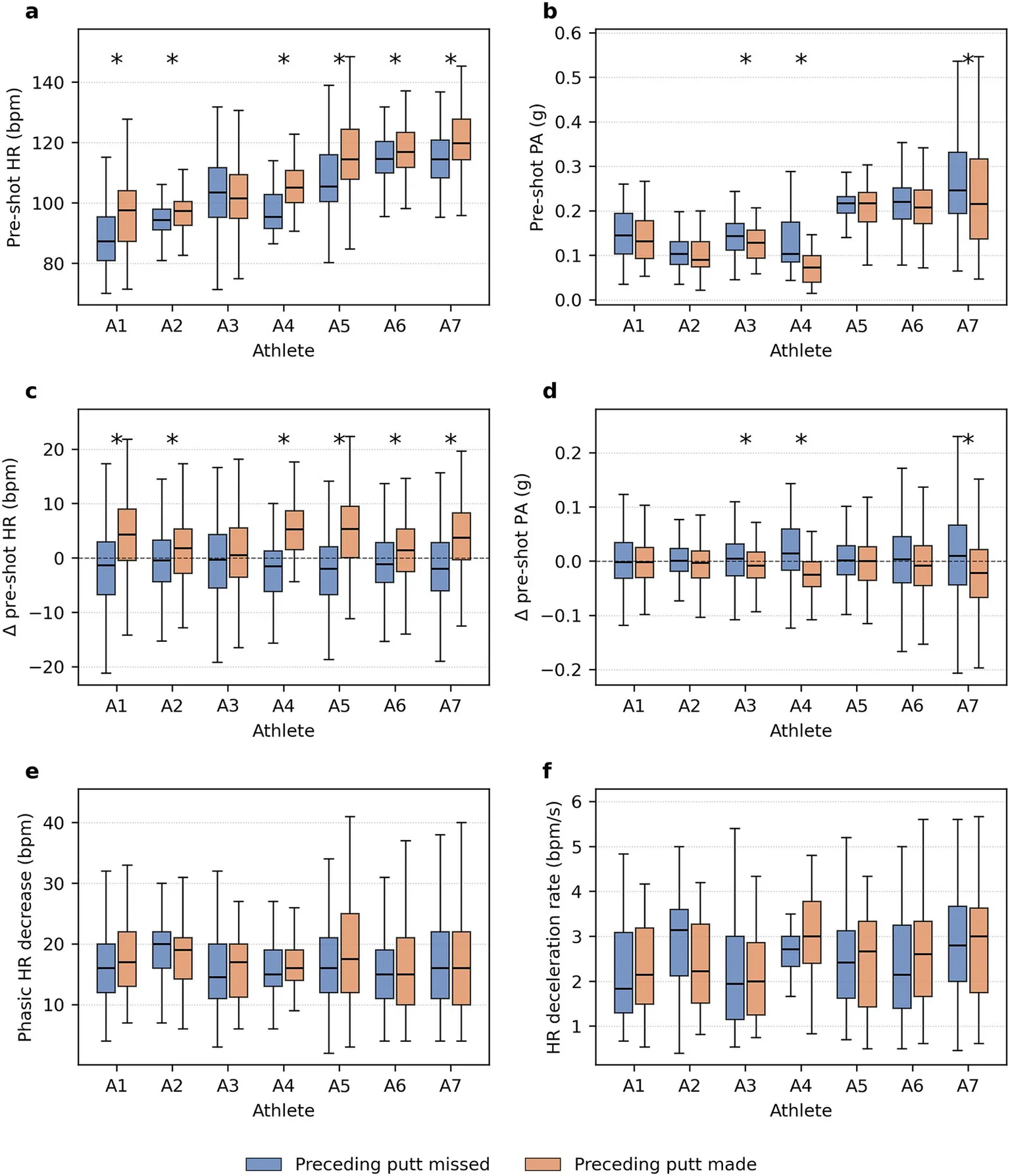
Distribution of the pre-shot HR (panel **a**), accelerometer-based PA levels (panel **b**), change in pre-shot HR (panel **c**), change in pre-shot PA (panel **d**), phasic HR decrease (panel **e**), and phasic HR deceleration rate (panel **f**) corresponding to whether the previous putt was missed or made, for each athlete (‘A1’–‘A7’). The distributions are shown as the median value and the interquartile range. The upper panels **(a,b)** show the absolute levels of HR and PA; the middle panels **(c,d)** show the change in HR and PA following missing or making the putt. A single asterisk marks a comparison that remains significant after Benjamini-Hochberg correction within the family of seven athlete-wise tests (Supplementary Tables S3, S4). No significant differences are observed for panels **(e,f)**.

In the two middle panels of Figures 3c,d, the data indicate how HR and PA change between consecutive putts. In other words, these results indicate how the pre-shot data changes after scoring or missing a putt. In line with the results in the two upper panels of Figure 3, the distributions indicate that pre-shot HR increases after scoring a putt and decreases after missing a putt. For six athletes, the increase in pre-shot HR after making a putt is significantly different from a zero median value (Wilcoxon signed rank test; FDR-adjusted *p* ≤ 0.026; median increase between 1.7 and 5.3 bpm), and for five athletes the decrease in pre-shot HR after missing the putt is significantly different from a zero median value (FDR-adjusted *p* ≤ 0.035; median decrease between 1.3 and 2.0 bpm). Under the more conservative Holm correction the decrease after missing remains significant for four of these five athletes, the exception being A6 (adjusted *p* = 0.074; Supplementary Table S4). Furthermore, the difference in pre-shot PA levels between consecutive putts is not as outspoken as the changes in HR. Three athletes record significantly decreased activity levels after making a putt, and for two of them the activity increases significantly after missing the putt. Finally, the two lower panels of Figures 3e,f, display the pre-shot HR decrease and pre-shot HR deceleration rate when matched with the outcome of the preceding putt. These data show that no significant differences are observed between the labels of made and missed putts, and all corresponding effect sizes are small (|r| ≤ 0.24).

Pooling all athletes in the mixed-effects models confirms this picture while accounting for the nesting of putts within sessions and athletes. The pre-shot HR is 5.08 bpm higher after a made putt than after a missed putt (95% CI [4.54, 5.62], t(3448) = 18.4, *p* < 0.001), and the change in pre-shot HR between consecutive putts is 4.88 bpm larger after a made putt (95% CI [4.33, 5.42], *p* < 0.001). Adding the pre-shot HR of the preceding putt as a covariate leaves this essentially unchanged (4.97 bpm, 95% CI [4.51, 5.44], *p* < 0.001), which indicates that the effect is not an artefact of the serial dependence between consecutive attempts. For PA the pooled estimate is a decrease of 0.016 g after a made putt (95% CI [−0.021, −0.012], *p* < 0.001), which is an order of magnitude smaller relative to the observed spread than the HR effect. Between-athlete and between-session differences account for a substantial part of the total variance in pre-shot HR (intraclass correlations of 0.22 and 0.54 respectively), which is precisely why these terms are modelled explicitly rather than ignored (Supplementary Table S5). Figure 4 summarises the athlete-wise effect sizes and the mixed-model estimates side by side.

**Figure 4:**
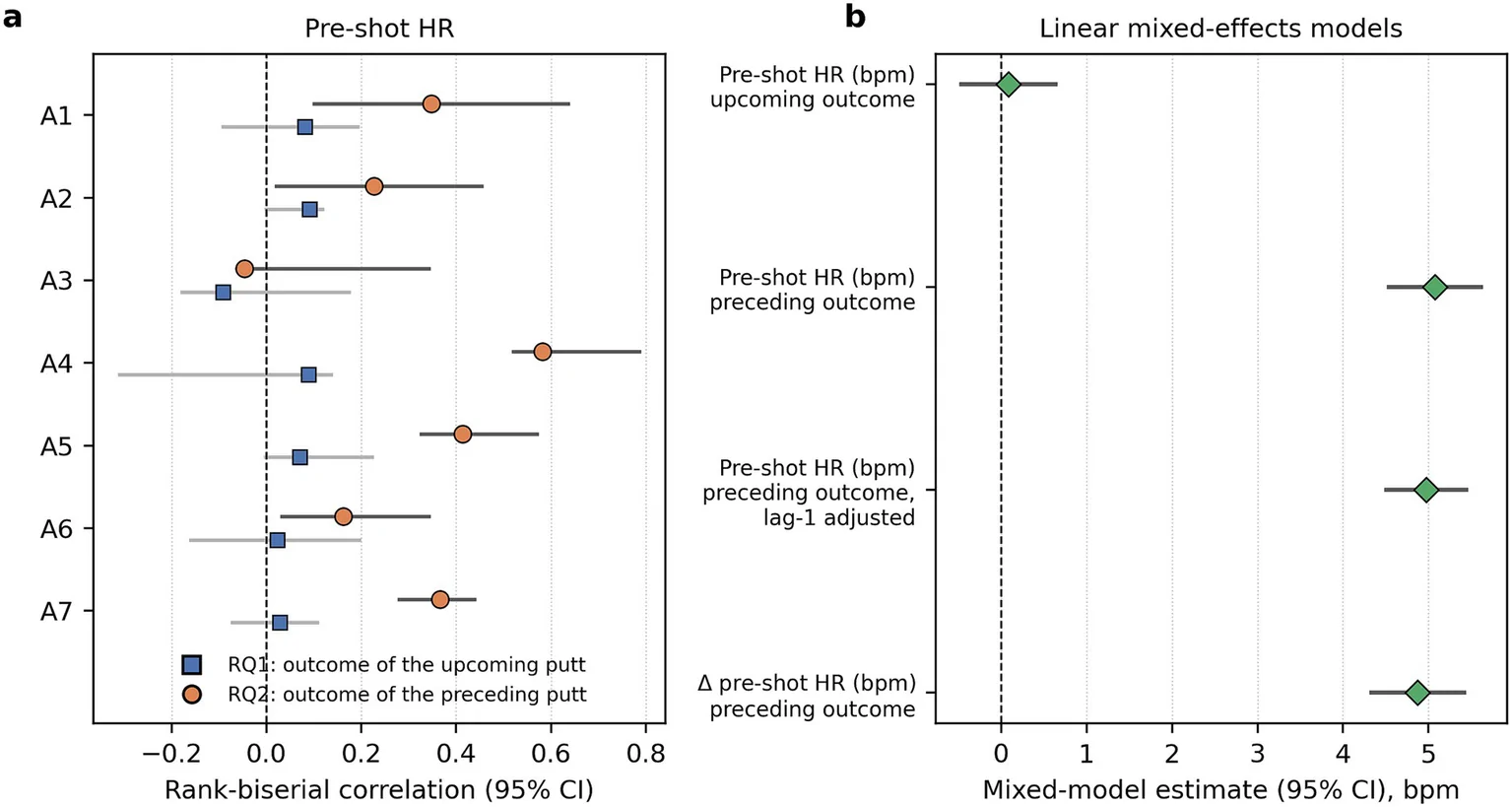
Effect sizes and pooled model estimates for the pre-shot HR. Panel **a**: rank-biserial correlations with 95% cluster-bootstrap confidence intervals for each athlete, comparing pre-shot HR between made and missed putts for the upcoming putt (squares, research question 1) and for the preceding putt (circles, research question 2). Panel **b**: fixed-effect estimates with 95% confidence intervals from the linear mixed-effects models with random intercepts for athlete and for session within athlete, expressed in beats per minute. The lag-1 adjusted model additionally includes the pre-shot HR of the preceding putt as a mean-centred covariate. The underlying values are given in Supplementary Tables S2, S3, S5.

In addition to the analysis of the pre-shot HR and PA levels with respect to the binary labels of making or missing a putt, Figure 5 displays the same data as Figure 3, but now using the labels of the current score while attempting to make 5 putts in a row. These results are included to provide additional insights in the idea that pressure increases after holing putts. Kruskal-Wallis testing for significant effects of the score on the distribution of pre-shot data is performed. For the pre-shot HR (panel a) and for the change in pre-shot HR (panel c), the association with the score is significant after FDR correction for six and for seven of the seven athletes respectively, with epsilon-squared values up to 0.34. The exception in panel a is A3 (H(4) = 4.1, *p* = 0.39). Because the number of observations decreases as the score increases by design, the per-score sample sizes range from 78 to 636 putts at a score of 0 to between 2 and 13 putts at a score of 4 (Supplementary Table S6). Dunn’s post-hoc tests with Holm correction show that the significant omnibus results are driven almost entirely by the contrast between a score of 0 and any higher score - for A5, for example, z ranges from −5.3 to −5.8 with adjusted *p* < 0.001 for all four comparisons against a score of 0 - whereas the contrasts among scores of 1 and above are mostly not significant (Supplementary Table S7). These analyses by score are exploratory, and the omnibus test used here compares the score levels without taking their ordering into account: they establish that the distribution of pre-shot HR differs across score levels, but not that it increases monotonically with the score. Given the small samples at the highest scores, differences involving a score of 3 or 4 should be interpreted with corresponding caution.

**Figure 5:**
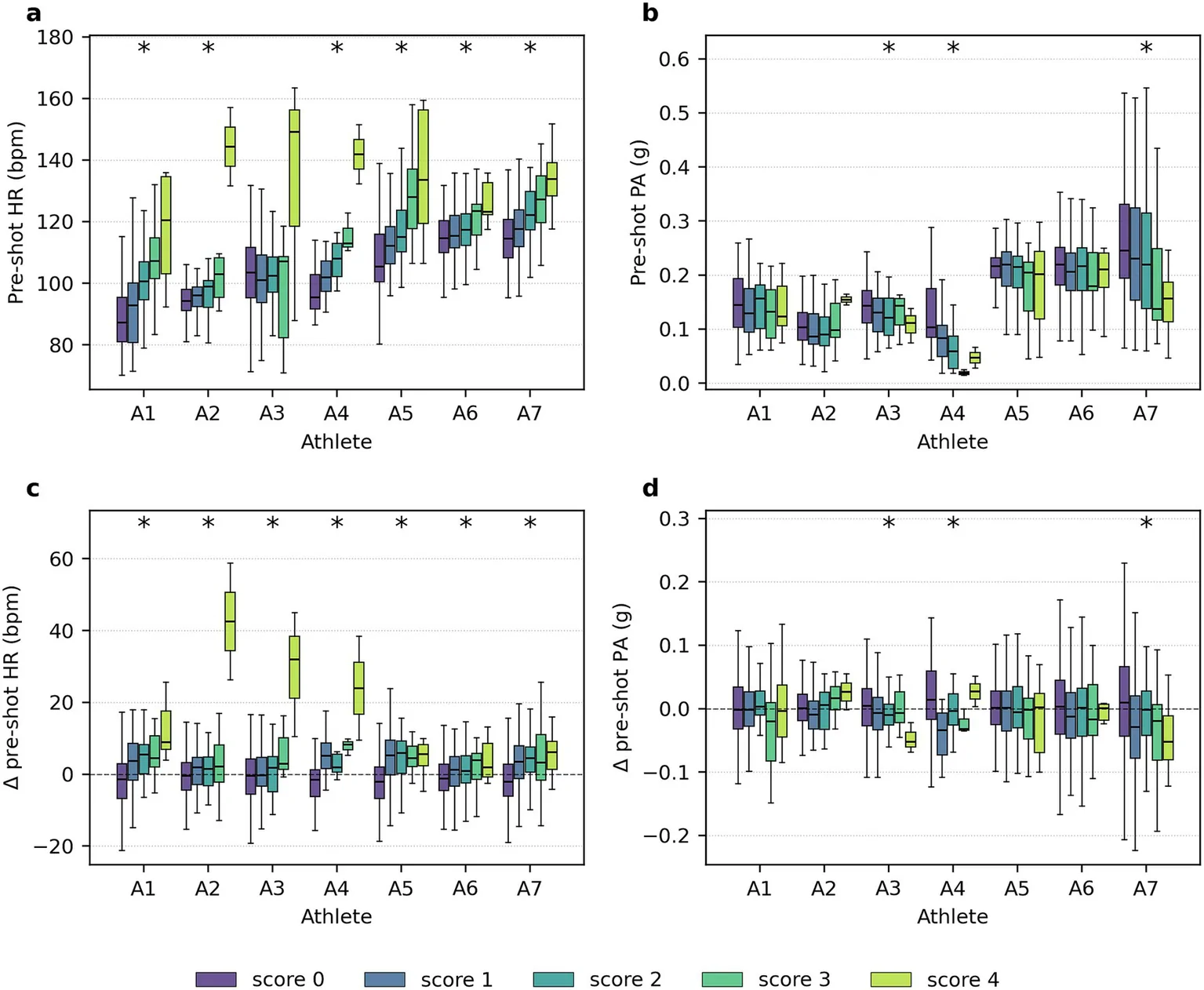
Distribution of the pre-shot HR (panel **a**), accelerometer-based PA levels (panel **b**), change in pre-shot HR (panel **c**), and change in pre-shot PA (panel **d**) corresponding to the score (0–4) resulting from the previous putt, for each athlete (‘A1’–‘A7’). The distributions are shown as the median value and the interquartile range. An asterisk marks a Kruskal-Wallis omnibus test that remains significant after Benjamini-Hochberg correction. The number of observations decreases as the score increases because of the design of the exercise; per-score sample sizes are given in Supplementary Table S6 and Holm-corrected Dunn post-hoc comparisons in Supplementary Table S7. The analysis by score is exploratory, and the tests reported here compare score levels without taking their ordering into account.

Finally, to present the reader with insight in the intra-individual variability of the pre-shot HR data, Figure 6 presents the same data used in Figure 3 panel c, but now for every individual putting session. Shading is added to this figure to distinguish between the sessions of different athletes. For 17 out of 29 measurements, the pre-shot HR significantly increases from zero subsequent to scoring a putt (16 out of 29 after FDR correction). Additionally, for 13 of these sessions, the pre-shot HR also significantly decreases from zero subsequent to missing a putt (8 out of 29 after FDR correction). The direction of the effect is nevertheless consistent well beyond the sessions that reach significance: the median change in pre-shot HR is positive after a made putt in 26 of the 29 sessions and negative after a missed putt in 26 of the 29 sessions, which indicates that the sessions failing to reach significance are predominantly the shorter ones rather than sessions showing the opposite pattern. As a further description of intra-individual variability, the within-session coefficient of variation of the pre-shot HR has a median of 6.6% (range 4.5 to 11.3%), while the intraclass correlations reported above indicate that 22% of the variance in pre-shot HR is attributable to stable differences between athletes and a further 54% to differences between sessions of the same athlete (Supplementary Table S8). These indices describe the variance structure of the data and its within- and between-session variability; they are not a formal reliability assessment.

**Figure 6:**
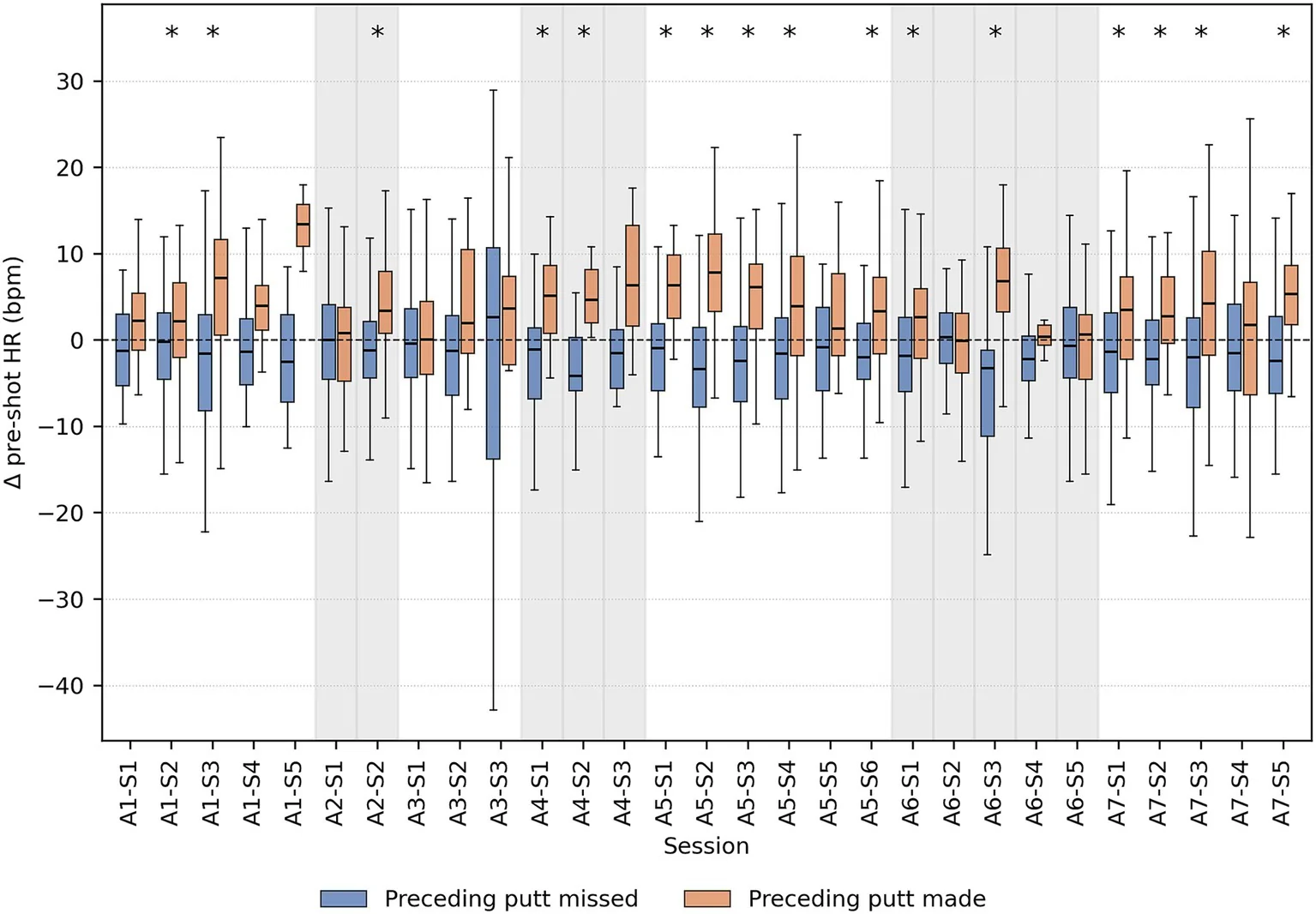
Distribution of the change in pre-shot HR corresponding to whether the previous putt was ‘Missed’ or ‘Made’, for each individual test session of each athlete (‘A1-S1’–‘A7-S5’). The distributions are shown as the median value and the interquartile range. Shading distinguishes the sessions of different athletes. An asterisk marks a session in which the increase after a made putt remains significantly different from a zero median (Wilcoxon signed rank test) after Benjamini-Hochberg correction across the 29 sessions (Supplementary Table S8).

## 4 Discussion

The first analyses consider how the HR and PA levels are distributed when linking the pre-shot data with the subsequent outcome for each putt. With this analysis, it is tested whether pre-shot data can be predictive for putting performance. Four different metrics are considered: the average pre-shot HR level, the average pre-shot PA level, the phasic HR decrease, and the HR deceleration rate. For only one athlete, the distribution of the average pre-shot PA levels is significantly different when comparing made and missed putts. For all other metrics and athletes, no significant differences are observed. These results are somewhat in conflict with previous studies that consider mental load during golf putting. For instance, monitoring QE and brain activity has shown to be able to distinguish between successful and failed putts based on pre-shot routine data (Sarig et al., 2023; Vickers, 1992). Moreover, the phasic HR decrease and deceleration rate have also been indicative for task difficulty, skill level and thus performance in golf putting (Boutcher and Zinsser, 1990; Cooke et al., 2014; Henderson et al., 2024; Neumann and Thomas, 2009, 2011). It is however important to note that the aforementioned studies deal with a laboratory setting in which participants performed a limited number of putts in specific and controlled conditions. As our results are obtained from a real-world golf setting in which athletes performed the putting exercise at their own pace, much more variability in the conditions of executing the task can be assumed. Given that the results of this study do not show a relation between the pre-shot state and the putting performance, the confounding factors that are inherently related to real-life conditions may affect such predictive analysis of performance. This null result is supported by an interval estimate rather than only by the absence of significance: the pooled mixed-effects model excludes any pre-shot HR difference larger than about 0.6 bpm between putts that will be made and putts that will be missed.

In line with the second research question, it is analysed how the HR and PA data are distributed when matching the outcome for each putt with the subsequent pre-shot routine. In this way, it is tested whether the outcome of a given putt is associated with the mental load of the next shot that will be taken. In terms of absolute values, the pre-shot HR is significantly higher after scoring a putt for all but one athlete. Although interesting, these data do not indicate whether the observed difference is related to a significant increase in HR after scoring or a significant decrease after missing, or perhaps both. Therefore, the change in pre-shot HR between consecutive putts is determined, and we note that for five athletes both a significant increase after scoring and a significant decrease after missing are occurring. The increase after scoring is most outspoken, with the largest values recorded. Furthermore, for 17 individual sessions (see Figure 6), this significant pattern in the changing HR responses is confirmed. The pooled mixed-effects estimate of 5.1 bpm (95% CI [4.5, 5.6]) quantifies the magnitude of this shift while accounting for the clustering of putts within sessions and athletes, and the fact that it barely changes when the preceding pre-shot HR is added as a covariate indicates that it is not an artefact of the serial dependence between consecutive attempts. When considering the phasic HR decrease and deceleration rate, no significant differences are observed. Before discussing what these data imply regarding mental load monitoring, let us evaluate the activity data in the same way. Given that each putt is performed from the same distance to the hole, a broadly comparable level of physical effort is required to take the shot. Surprisingly, the PA levels after scoring are significantly lower for three athletes. For three more athletes, the median PA value is also lower, yet not statistically significant. Analyses of the changes in pre-shot PA show that a decrease after scoring is most outspoken.

These observations of changing HR and PA data following putting performance are consistent with an interpretation in terms of the mental load experienced by the youth athletes during these putting sessions, but, as set out below, they do not establish it. As mentioned in the introduction, mental load is defined as increases in the cognitive demands of a task (i.e., putting), which entails a rise in the recruitment of processing resources (Shuggi et al., 2017). The observed HR increases following successful putts and decreases after missed attempts align with established theories linking cardiovascular responses to task difficulty and arousal (Shuggi et al., 2017). Unlike laboratory-based methods such as EEG or gaze tracking, HR offers a practical approach for understanding how athletes physiologically adapt to pressure situations in real-world environments. The absence of corresponding PA changes is consistent with, though not proof of, HR reflecting mental rather than physical load. Together, these findings support the use of HR and PA as accessible signals for exploring cognitive demands in field conditions, where controlled experimental setups are often unfeasible. Mental load is only one of many factors that influence HR and PA. Several arguments that make a mental-load interpretation plausible are discussed hereafter; individually and together, they render that interpretation reasonable rather than establish it. First, the fact that changing HR data do not correspond to similar changes in PA set off the interpretation that HR can provide a window into the mental load. During regular, light-to-moderate physical exercise, a quasi-proportional relation between PA and HR is common knowledge (Bunc et al., 1988). Even though putting is a task that requires limited physical efforts (Carless and Douglas, 2004), coincidental increases in PA and HR would have made it difficult (or perhaps even impossible) to attribute a portion of the HR response to factors like mental load. In short, the opposing trend in both variables suggests that PA is not the driving factor for the HR changes in this particular scenario. Secondly, since all putts require the same level of physical effort, the design of only completing the task when scoring five consecutive shots is thought to induce mental load rather than physical load by creating pressure situations. Carr (2015) defines such situations as unusual occurrences fraught with extra value, placing demands above what has commonly been experienced, especially motivational and emotional demands (Carr, 2015). Putts from 3-metre distance to the hole have a success rate of about 41% for professional golfers (Gryc et al., 2021). Having to score five times in a row thus inevitably creates ‘all or nothing’ situations. Third, the experimental set-up was designed to reasonably rule out other dominant processes of significantly affecting HR, such as changing environmental conditions, food intake, sickness, etc. This is because each session was performed in a relatively short duration (< 2 h of light physical efforts) in a stable outdoor environment. Participants were allowed to drink water, and could perform the putting exercise at their own pace. Fourth, the exploratory analysis presented in Figure 5 indicates that the score, rather than only the binary labels of ‘Made’ and ‘Missed’, is also associated with graded differences in HR. As athletes make a series of consecutive successful putts, the pre-shot HR levels are higher at higher scores. We did not test formally whether this increase is monotonic, and the number of observations at the highest scores is small, so this observation should be read as suggestive rather than as an established trend.

Nevertheless, the argumentation should not be pushed too far. A chest-mounted accelerometer captures gross trunk movement and is a robust indicator of whether an athlete is walking, standing or bending, but it is comparatively insensitive to the small postural adjustments, the isometric grip and forearm activity, and the balance corrections that accompany a putting stroke, and it is not a measure of muscular effort. Between attempts the athlete also walks to a different marker and retrieves the ball. Although the markers were arranged so that these walks were short and of comparable length regardless of the outcome, walking distance and pace were not measured. A systematic difference in how briskly an athlete walks after a success as opposed to after a miss (which is itself plausible on motivational grounds) could produce the kind of HR difference we observe, and would be only partly reflected in a chest-worn accelerometer signal. The dissociation between HR and PA is therefore evidence against a simple, whole-body physical-effort explanation, but it is not evidence that no motor contribution exists.

In addition to mental load, other processes and mechanisms may be considered for attributing the observed patterns. For instance, the underlying process could be affective rather than cognitive: holing a putt is a rewarding event and missing one is a mildly aversive one, so the HR rise after a success and the fall after a miss may track short-lived, reward-related arousal and its valence rather than any change in the recruitment of processing resources. Core-affect models treat arousal and valence as dimensions that are separable from cognitive demand (Russell and Barrett, 1999), and the definition of mental load adopted in the Introduction (i.e., a rise in the recruitment of processing resources; Shuggi et al., 2017) is not the same construct as excitement following success. A second account is motivational: as the streak lengthens, the incentive value of the next putt rises, which is the classical definition of a pressure situation (Baumeister, 1984). A third possibility is attentional: after a success the athlete may shift towards a more self-focused, explicitly monitored execution of a movement that is normally automatic (Carr, 2015; Eysenck et al., 2007). A fourth is methodological, namely that a made and a missed putt are followed by slightly different sequences of physical actions, an issue we return to below. Importantly, the higher pre-shot HR at higher running scores (Figure 5), which we did not test formally for monotonicity, is compatible with the mental-load, the motivational and the affective accounts alike, and therefore does not discriminate between them. Disentangling these accounts requires measures that the present study did not collect. We therefore present the link with mental load as the interpretation that motivated the study and that is consistent with the data, not as the interpretation that the data select.

In addition to these insights in how HR and PA during pre-shot routines vary with the preceding performance in real-life training sessions, the main added value of this study is deemed to lie in its practical application. This set-up and methodology may give athletes and coaches a preliminary, exploratory way to start looking at how performance and pre-shot physiological states are linked with each other during real-world training. In the absence of subjective mental-load measures, this remains a hypothesis-generating aid rather than a validated diagnostic tool. In itself, the changing HR and PA data do not reflect the quality/efficiency of the training process. Rather, by flagging candidate shifts in physiological state that may be related to mental load, these results could provide an exploratory conversation starter between coach and athlete about how certain sessions or conditions are perceived. As shown in Figure 6, not every session for a certain athlete yields significant differences. By means of such data, coaches and athletes can start unravelling why certain sessions bear this added mental load and others do not. Having such data provides a common ground for discussion, allowing everyone to reflect on the same effects of the training setting instead of working with intangible concepts like stress or mental load, which may be difficult to communicate. Especially when working with youth athletes, this may help them understand their own training responses.

It is important to be specific about what this study does and does not show on this point. What the data establish is a reproducible, within-athlete association between the outcome of a putt and the cardiac state during the preparation of the next, of a magnitude that survives a statistical treatment appropriate to the clustered design. In the current study, no feedback was delivered to the athletes or to their coaches at any moment during the measurements, no self-regulation outcome was assessed, and no intervention was evaluated. Therefore, we state our findings as such: wearable monitoring of this kind may support future self-regulation interventions, and the present results indicate that the signal such an intervention would rely on is measurable in an ordinary training session. Whether the feedback improves self-regulation, skill learning or transfer to competition is an open question that this observational design cannot resolve.

### 4.1 Limitations and implications

The interpretation of these findings is constrained by a number of limitations. First, and most importantly, no subjective reference data on the perceived mental load were collected, so our physiological interpretation cannot be validated against the athletes’ own experience. Validated instruments for this purpose could be the NASA Task Load Index (Hart and Staveland, 1988), a single-item mental effort or pressure rating, or the CSAI-2 (Martens et al., 1990) administered at appropriate time points. This was a deliberate design decision rather than a matter of participant refusal: administering such instruments between putts would have disrupted the flow of a self-paced, real-world training exercise with young athletes, and the burden on the participants was a primary concern of the collaborating programme. We nevertheless acknowledge that their absence substantially weakens the mental-load claim, and that a brief single-item rating of effort together with a brief rating of valence and arousal, both of which take a few seconds to collect, would be the single most valuable addition to a replication. Until such data exist, the HR- and PA-based indices reported here should be treated as a potential indirect correlate of mental load and not as a measure of it.

Second, some statistical limitations should be stated plainly. The mixed-effects models presented here account for the clustering of putts within sessions and athletes, but accounting for clustered observations does not overcome the limited population-level sample. The 3,691 putts increase the precision with which the response of each individual athlete is estimated; they do not increase the number of individuals contributing to a population-level inference, and seven athletes therefore remain the effective unit of generalisation however many putts each of them contributed. The confidence intervals reported for the pooled estimates are conditional on the seven athletes observed, and a random-intercept structure absorbs the mean differences between athletes and sessions without modelling the full autocorrelation structure of consecutive putts; the lag-1 adjusted models address this only to first order. A study designed to generalise across athletes rather than to describe these athletes would require a substantially larger and prospectively sampled cohort.

Third, the sample was limited to the seven skilled youth golfers available within the collaborating programme, and their standard was heterogeneous (handicap index ≤ 4 for four athletes and ≤ 12 for three). This modest, heterogeneous convenience sample limits generalisability and complicates between-athlete comparison. The group was also predominantly male (six males, one female), which precludes any inference about sex differences. While the sample size allowed for detailed individual analyses, the generalisability of the findings to a broader population is constrained. The number of sessions per athlete varied as well, because availability was influenced by illness, school schedules and other personal circumstances, and this variability in session count and timing may have influenced individual response patterns.

Fourth, the ecological validity of this design comes at the price of experimental control. The sessions were conducted outdoors on a practice green, where weather, temperature, wind and lighting were not controlled; sessions took place at different times of day, so circadian variation in resting heart rate is not accounted for; fatigue accumulates within a session and across a training week; motivation, coach interactions and the presence of team-mates varied naturally; and nutrition, hydration and sleep were not recorded. Each of these can influence heart rate, in several cases by more than the difference reported here. Two features of the design provide partial protection. The contrast of interest is made within an athlete and, for the change scores and the session-level analyses, within a session and between consecutive putts separated by less than a minute, so slow-acting sources of variation such as weather or circadian phase are largely differenced out. In addition, the random intercepts for session absorb any factor that is constant within a session. What remains unprotected is any factor that varies within a session and is systematically associated with the outcome of a putt, and the affective and motivational accounts discussed above are exactly of that kind.

Fifth, the pre-shot window length of 5 s is inspired by the literature (Crews and Boutcher, 1986), but it remains a choice rather than a derived quantity, and even a 15-s window contains relatively few 1 Hz samples so that the influence of individual outlying samples cannot be discounted. The analyses were repeated with 10- and 15-s windows using identical models and corrections (Supplementary Table S1). The direction and significance of the HR findings are preserved across all three windows, although the magnitude of the effect attenuates as the window lengthens (from 5.08 bpm at 5 s to 3.06 bpm at 15 s), which is consistent with a response that is concentrated in the seconds immediately before ball contact. The PA findings are not robust in this way: the pooled PA effect reverses sign between the 5-s and the 15-s window, which is itself informative because it suggests that longer windows increasingly capture the walk to the marker rather than the pre-shot phase proper. PA results should therefore be read as window-dependent and treated with more caution than the HR results.

Sixth, the accelerometer data are recorded on the upper body. This provides a robust but non-specific measure of physical activity, as discussed above. Recording accelerations at the arm or wrist would provide additional information on the swing of the putter itself, and measurements of muscle activation or of the pressure applied to the grip would be golf-specific alternatives. These require specialised equipment that may affect the athletes’ behaviour or is simply less practical for daily use, which is why the chest strap was deemed an acceptable compromise; but the compromise does place a real limit on how strongly the HR/PA dissociation can be interpreted.

Seventh, by the nature of the set-up the number of observations at the fourth and fifth consecutive putt is small. Per-score sample sizes are reported in Supplementary Table S6, and the Holm-corrected Dunn post-hoc tests (Supplementary Table S7) show that the association with the score is carried mainly by the contrast between a score of zero and any higher score. Comparisons involving the highest scores rest on as few as two observations for some athletes and should not be relied upon.

Finally, putting is only one part of the game of golf. When playing actual rounds, the PA due to walking and hitting longer shots will affect HR, and the proposed methodology is currently limited to the specific context of putting exercises during training. A particularly valuable extension would be to capture genuinely ‘clutch’ putts during competitive rounds, where holing or missing carries real consequences for the gross score; such situations would impose an ecologically meaningful pressure that a training drill can only approximate. We also emphasise that the ‘five-in-a-row’ requirement is intended to create self-imposed pressure, but the outcome carried no competitive consequence, and the results should be read with that distinction in mind.

Despite these limitations, the findings illustrate the potential of wearable technology for unobtrusive physiological monitoring in real-world sports settings, and for generating hypotheses about mental load that can subsequently be tested with appropriate subjective measures. By using heart rate and physical activity data, this study highlights the dynamic interplay between physiological responses and performance outcomes in the natural training environment of athletes. These insights provide a foundation for further research aimed at optimising training strategies and fostering athlete development. Future studies may benefit from incorporating larger and more diverse samples, controlling environmental conditions more rigorously, and combining physiological with concurrent subjective measures to enhance the understanding of mental load in sports contexts.

## 5 Conclusion

This study set out to explore whether heart rate (HR) and physical activity (PA), recorded with an unobtrusive chest-worn wearable, can serve as a potential, indirect correlate of the mental load experienced by youth golfers during putting practice. Across 3,691 putts collected in a real training environment, pre-shot HR and PA did not distinguish made from missed putts, and the pooled mixed-effects estimate is precise enough to exclude any difference larger than about 0.6 bpm. The pre-shot state of the next attempt did, however, differ systematically according to the outcome of the preceding putt: for the majority of athletes, HR was higher following a made putt and lower following a missed one, while PA showed the opposite, weaker pattern. Pooled over athletes and sessions, and after accounting for the nesting of putts within sessions and athletes, this amounts to a difference of 5.1 bpm (95% CI [4.5, 5.6]). This dissociation between the two signals makes a mental- rather than physical-load reading of the HR response one plausible interpretation, but not the only one: an affective, reward-related account of the same pattern remains equally compatible with these data, and the chest-worn accelerometer cannot exclude a residual motor contribution. In the absence of concurrent subjective ratings, this remains an interpretation to be validated rather than an established measure.

Positioned within the wider use of information and communication technologies (ICT) for athlete development, the novelty of this work lies in its ecological implementation and its potential coaching application rather than in heart rate monitoring itself, which is long established in this literature. What is new is that an accessible, low-cost sensor renders an otherwise invisible physiological signal, and with it a potential correlate of mental load, available *in situ*, across thousands of putts of ordinary training, and that the resulting signal is large and consistent enough to survive an analysis that respects the clustered structure of the data. Whether that signal can be turned into useful feedback is the obvious next question, and it is one this study poses rather than answers: it may help scaffold the self-regulation and metacognitive awareness that distinguish more- from less-successful youth athletes (Jonker et al., 2010), but demonstrating that requires an intervention study in which feedback is actually delivered and a self-regulation outcome is actually measured.

## Data Availability

The datasets presented in this article are not readily available because the data that support the findings of this study are available on reasonable request from the corresponding author. The data are not publicly available due to privacy measures that relate to the limited size of the test population. Requests to access the datasets should be directed to jasper.gielen@kuleuven.be.
