# Supplementary material for: Wearable monitoring of pre-shot heart rate in youth golfers: a potential mental-load correlate in ecological putting practice
Source: Front Psychol. 2026 Oct 28;17:1939721. doi: 10.3389/fpsyg.2026.1939721 (PMC13752978; doi:10.3389/fpsyg.2026.1939721)
Supplement: Supplementary file 1 [file Data_Sheet_1.DOCX]

Supplementary Material

**Wearable heart rate as an exploratory correlate of mental load during youth golf putting practice: an observational field study**

Jasper Gielen, Katrien Buysse, Elise Heirman, Romain Meeusen, Jean-Marie Aerts

This document reports the analyses that support, but are not central to, the results presented in the manuscript: the sensitivity of the findings to the length of the pre-shot window (Section S1), the complete athlete-wise test statistics with effect sizes and multiplicity-corrected p-values (Section S2), the full output of the linear mixed-effects models (Section S3), the score-level analyses with per-score sample sizes and post-hoc comparisons (Section S4), and the session-level results (Section S5).

Unless stated otherwise, r denotes the rank-biserial correlation, 95% confidence intervals for effect sizes were obtained by a cluster bootstrap resampling whole sessions, and p (Holm) and p (FDR) denote p-values corrected within each family of seven athlete-wise tests using the Holm–Bonferroni and Benjamini–Hochberg procedures respectively. A dash indicates that a test could not be computed because fewer than the required number of observations were available.

# S1. Sensitivity to the length of the pre-shot window

The 5-second pre-shot window used in the manuscript follows the definition of the pre-shot phase given by Crews and Boutcher (1986). To establish how far the findings depend on that choice, all analyses were repeated for pre-shot windows of 10 and 15 seconds using identical models, effect sizes and corrections for multiple comparisons.

The heart-rate findings are robust to the window length. The null result for the first research question holds at every window length, and the effect of the preceding outcome on the pre-shot HR is significant at every window length. Its magnitude attenuates as the window lengthens, from 5.08 bpm at 5 s to 4.05 bpm at 10 s and 3.06 bpm at 15 s, which is what would be expected of a response concentrated in the seconds immediately preceding ball contact and progressively diluted by earlier, less task-related seconds.

The physical-activity findings are not robust in this way. The pooled PA effect of the preceding outcome is negative at 5 s (-0.016 g), slightly positive at 10 s (+0.006 g) and clearly positive at 15 s (+0.018 g). The most plausible explanation for this reversal is that longer windows increasingly capture the walk from the previous marker rather than the pre-shot phase proper. PA results should therefore be read as window-dependent and treated with more caution than the HR results.

**Table S1. Sensitivity of the mixed-effects results to the length of the pre-shot window. Estimates are in bpm for HR and in gravitational units for PA. All models include random intercepts for athlete and for session within athlete. The final column gives the number of athletes for whom the corresponding athlete-wise Wilcoxon test survives Benjamini–Hochberg correction.**

| **Window** | **Model** | **Estimate** | **95% CI** | **p** | **Athletes significant (FDR)** |
| --- | --- | --- | --- | --- | --- |
| 5 s | Pre-shot HR ~ outcome of the upcoming putt | +0.086 | [-0.465, +0.636] | 0.760 | 0/7 |
| 5 s | Pre-shot HR ~ outcome of the preceding putt | +5.077 | [+4.538, +5.617] | <0.001 | 6/7 |
| 5 s | Change in pre-shot HR ~ outcome of the preceding putt | +4.875 | [+4.334, +5.416] | <0.001 | 6/7 |
| 5 s | Pre-shot PA ~ outcome of the preceding putt | -0.016 | [-0.021, -0.012] | <0.001 | 3/7 |
| 10 s | Pre-shot HR ~ outcome of the upcoming putt | +0.072 | [-0.430, +0.574] | 0.778 | 0/7 |
| 10 s | Pre-shot HR ~ outcome of the preceding putt | +4.052 | [+3.552, +4.552] | <0.001 | 5/7 |
| 10 s | Change in pre-shot HR ~ outcome of the preceding putt | +3.901 | [+3.411, +4.391] | <0.001 | 7/7 |
| 10 s | Pre-shot PA ~ outcome of the preceding putt | +0.006 | [+0.003, +0.010] | <0.001 | 4/7 |
| 15 s | Pre-shot HR ~ outcome of the upcoming putt | -0.122 | [-0.599, +0.355] | 0.616 | 0/7 |
| 15 s | Pre-shot HR ~ outcome of the preceding putt | +3.058 | [+2.574, +3.541] | <0.001 | 5/7 |
| 15 s | Change in pre-shot HR ~ outcome of the preceding putt | +3.159 | [+2.723, +3.596] | <0.001 | 6/7 |
| 15 s | Pre-shot PA ~ outcome of the preceding putt | +0.018 | [+0.015, +0.021] | <0.001 | 5/7 |

Figure S1. Fixed-effect estimates with 95% confidence intervals from the linear mixed-effects models, for pre-shot windows of 5, 10 and 15 seconds. Left: effect of the outcome of the upcoming putt on pre-shot HR (bpm). Centre: effect of the outcome of the preceding putt on pre-shot HR (bpm). Right: effect of the outcome of the preceding putt on pre-shot PA (g).

# S2. Athlete-wise results

The tables in this section give the complete within-athlete test statistics summarised in Figures 2, 3 and 6 of the manuscript. Tables S2 and S3 report the two-sample comparisons for the two research questions; Table S4 reports the one-sample tests of the change scores against a zero median.

**Table S2. Pre-shot metrics compared between putts that were subsequently made and putts that were subsequently missed (Wilcoxon rank sum test), for each athlete.**

| **Metric** | **Athlete** | **n made** | **n missed** | **Median made** | **Median missed** | **z** | **p** | **p (Holm)** | **p (FDR)** | **r** | **95% CI** |
| --- | --- | --- | --- | --- | --- | --- | --- | --- | --- | --- | --- |
| Pre-shot HR (bpm) | A1 | 147 | 219 | 93.167 | 89.167 | 1.31 | 0.191 | 0.953 | 0.373 | 0.081 | [-0.091, 0.193] |
| Pre-shot HR (bpm) | A2 | 101 | 163 | 95.833 | 94.667 | 1.25 | 0.213 | 0.953 | 0.373 | 0.091 | [-0.001, 0.118] |
| Pre-shot HR (bpm) | A3 | 156 | 373 | 101.833 | 103.333 | -1.65 | 0.098 | 0.687 | 0.373 | -0.091 | [-0.178, 0.174] |
| Pre-shot HR (bpm) | A4 | 47 | 76 | 101.167 | 97.917 | 0.82 | 0.412 | 1.000 | 0.548 | 0.088 | [-0.310, 0.136] |
| Pre-shot HR (bpm) | A5 | 234 | 454 | 109.083 | 107.917 | 1.50 | 0.133 | 0.798 | 0.373 | 0.070 | [-0.003, 0.222] |
| Pre-shot HR (bpm) | A6 | 194 | 306 | 115.667 | 115.583 | 0.42 | 0.673 | 1.000 | 0.673 | 0.022 | [-0.160, 0.195] |
| Pre-shot HR (bpm) | A7 | 349 | 632 | 116.833 | 116.667 | 0.72 | 0.470 | 1.000 | 0.548 | 0.028 | [-0.073, 0.106] |
| Pre-shot PA (g) | A1 | 147 | 219 | 0.153 | 0.135 | 0.85 | 0.394 | 1.000 | 0.919 | 0.053 | [-0.063, 0.125] |
| Pre-shot PA (g) | A2 | 102 | 169 | 0.101 | 0.098 | -0.37 | 0.709 | 1.000 | 0.941 | -0.027 | [-0.132, -0.027] |
| Pre-shot PA (g) | A3 | 167 | 384 | 0.140 | 0.138 | 0.29 | 0.772 | 1.000 | 0.941 | 0.016 | [-0.052, 0.055] |
| Pre-shot PA (g) | A4 | 47 | 76 | 0.088 | 0.098 | -1.40 | 0.161 | 0.964 | 0.562 | -0.151 | [-0.203, 0.343] |
| Pre-shot PA (g) | A5 | 291 | 578 | 0.218 | 0.217 | 0.11 | 0.911 | 1.000 | 0.941 | 0.005 | [-0.144, 0.079] |
| Pre-shot PA (g) | A6 | 194 | 306 | 0.214 | 0.215 | 0.07 | 0.941 | 1.000 | 0.941 | 0.004 | [-0.110, 0.114] |
| Pre-shot PA (g) | A7 | 349 | 632 | 0.247 | 0.230 | 2.04 | 0.041 | 0.289 | 0.289 | 0.079 | [-0.010, 0.161] |
| Phasic HR decrease (bpm) | A1 | 91 | 147 | 17.000 | 16.000 | 1.10 | 0.271 | 1.000 | 0.606 | 0.085 | [-0.071, 0.236] |
| Phasic HR decrease (bpm) | A2 | 54 | 88 | 18.000 | 19.000 | -0.64 | 0.519 | 1.000 | 0.606 | -0.064 | [-0.145, 0.101] |
| Phasic HR decrease (bpm) | A3 | 54 | 117 | 15.000 | 15.000 | 0.96 | 0.338 | 1.000 | 0.606 | 0.091 | [0.085, 0.500] |
| Phasic HR decrease (bpm) | A4 | 26 | 50 | 15.000 | 16.000 | -1.07 | 0.284 | 1.000 | 0.606 | -0.150 | [-0.600, -0.058] |
| Phasic HR decrease (bpm) | A5 | 103 | 194 | 17.000 | 16.000 | 0.22 | 0.827 | 1.000 | 0.827 | 0.015 | [-0.099, 0.099] |
| Phasic HR decrease (bpm) | A6 | 163 | 243 | 15.000 | 14.000 | 0.69 | 0.489 | 1.000 | 0.606 | 0.040 | [-0.089, 0.183] |
| Phasic HR decrease (bpm) | A7 | 173 | 339 | 17.000 | 16.000 | 0.65 | 0.514 | 1.000 | 0.606 | 0.035 | [-0.172, 0.066] |
| HR deceleration rate (bpm/s) | A1 | 54 | 101 | 2.311 | 2.000 | 1.60 | 0.110 | 0.767 | 0.463 | 0.156 | [-0.193, 0.488] |
| HR deceleration rate (bpm/s) | A2 | 27 | 45 | 2.143 | 3.111 | -1.29 | 0.199 | 1.000 | 0.463 | -0.182 | [-0.182, -0.108] |
| HR deceleration rate (bpm/s) | A3 | 41 | 88 | 1.857 | 2.000 | -0.73 | 0.468 | 1.000 | 0.571 | -0.080 | [-0.089, -0.057] |
| HR deceleration rate (bpm/s) | A4 | 9 | 18 | 2.800 | 2.657 | 0.93 | 0.354 | 1.000 | 0.571 | 0.222 | [-0.300, 0.600] |
| HR deceleration rate (bpm/s) | A5 | 31 | 80 | 2.333 | 2.586 | -1.30 | 0.195 | 1.000 | 0.463 | -0.159 | [-0.333, 0.089] |
| HR deceleration rate (bpm/s) | A6 | 104 | 146 | 2.450 | 2.000 | 0.69 | 0.489 | 1.000 | 0.571 | 0.051 | [-0.074, 0.408] |
| HR deceleration rate (bpm/s) | A7 | 99 | 184 | 3.000 | 2.732 | 0.56 | 0.575 | 1.000 | 0.575 | 0.040 | [-0.157, 0.094] |

**Table S3. Pre-shot metrics compared according to the outcome of the preceding putt (Wilcoxon rank sum test), for each athlete.**

| **Metric** | **Athlete** | **n made** | **n missed** | **Median made** | **Median missed** | **z** | **p** | **p (Holm)** | **p (FDR)** | **r** | **95% CI** |
| --- | --- | --- | --- | --- | --- | --- | --- | --- | --- | --- | --- |
| Pre-shot HR (bpm) | A1 | 148 | 223 | 97.583 | 87.333 | 5.67 | <0.001 | <0.001 | <0.001 | 0.347 | [0.101, 0.635] |
| Pre-shot HR (bpm) | A2 | 101 | 165 | 97.333 | 94.333 | 3.11 | 0.002 | 0.006 | 0.002 | 0.227 | [0.021, 0.454] |
| Pre-shot HR (bpm) | A3 | 158 | 374 | 101.583 | 103.500 | -0.84 | 0.401 | 0.401 | 0.401 | -0.046 | [-0.057, 0.342] |
| Pre-shot HR (bpm) | A4 | 48 | 78 | 105.083 | 95.417 | 5.47 | <0.001 | <0.001 | <0.001 | 0.581 | [0.520, 0.786] |
| Pre-shot HR (bpm) | A5 | 230 | 463 | 114.500 | 105.500 | 8.88 | <0.001 | <0.001 | <0.001 | 0.414 | [0.327, 0.569] |
| Pre-shot HR (bpm) | A6 | 196 | 309 | 116.917 | 114.667 | 3.08 | 0.002 | 0.006 | 0.002 | 0.162 | [0.032, 0.342] |
| Pre-shot HR (bpm) | A7 | 350 | 636 | 119.833 | 114.500 | 9.53 | <0.001 | <0.001 | <0.001 | 0.367 | [0.280, 0.438] |
| Pre-shot PA (g) | A1 | 148 | 223 | 0.132 | 0.145 | -1.41 | 0.158 | 0.316 | 0.185 | -0.087 | [-0.183, 0.056] |
| Pre-shot PA (g) | A2 | 103 | 170 | 0.090 | 0.103 | -1.74 | 0.081 | 0.294 | 0.113 | -0.126 | [-0.377, -0.027] |
| Pre-shot PA (g) | A3 | 167 | 387 | 0.128 | 0.143 | -3.60 | <0.001 | 0.002 | <0.001 | -0.193 | [-0.513, 0.071] |
| Pre-shot PA (g) | A4 | 48 | 78 | 0.073 | 0.103 | -4.98 | <0.001 | <0.001 | <0.001 | -0.530 | [-0.725, -0.253] |
| Pre-shot PA (g) | A5 | 291 | 584 | 0.217 | 0.217 | -0.13 | 0.898 | 0.898 | 0.898 | -0.005 | [-0.279, 0.129] |
| Pre-shot PA (g) | A6 | 196 | 309 | 0.207 | 0.220 | -1.79 | 0.074 | 0.294 | 0.113 | -0.094 | [-0.213, 0.060] |
| Pre-shot PA (g) | A7 | 350 | 636 | 0.216 | 0.246 | -4.29 | <0.001 | <0.001 | <0.001 | -0.165 | [-0.308, -0.091] |
| Change in pre-shot HR (bpm) | A1 | 148 | 223 | 4.333 | -1.333 | 7.29 | <0.001 | <0.001 | <0.001 | 0.447 | [0.284, 0.574] |
| Change in pre-shot HR (bpm) | A2 | 101 | 162 | 1.833 | -0.417 | 2.56 | 0.011 | 0.021 | 0.012 | 0.188 | [-0.023, 0.470] |
| Change in pre-shot HR (bpm) | A3 | 152 | 369 | 0.500 | -0.333 | 1.93 | 0.053 | 0.053 | 0.053 | 0.108 | [0.047, 0.373] |
| Change in pre-shot HR (bpm) | A4 | 48 | 78 | 5.250 | -1.500 | 6.45 | <0.001 | <0.001 | <0.001 | 0.685 | [0.627, 0.874] |
| Change in pre-shot HR (bpm) | A5 | 222 | 439 | 5.333 | -2.000 | 11.13 | <0.001 | <0.001 | <0.001 | 0.530 | [0.404, 0.626] |
| Change in pre-shot HR (bpm) | A6 | 196 | 309 | 1.417 | -1.167 | 4.03 | <0.001 | <0.001 | <0.001 | 0.213 | [-0.051, 0.465] |
| Change in pre-shot HR (bpm) | A7 | 350 | 636 | 3.750 | -2.000 | 11.54 | <0.001 | <0.001 | <0.001 | 0.444 | [0.320, 0.540] |
| Change in pre-shot PA (g) | A1 | 148 | 223 | -0.002 | -0.002 | -1.05 | 0.293 | 0.592 | 0.293 | -0.065 | [-0.100, 0.034] |
| Change in pre-shot PA (g) | A2 | 103 | 170 | -0.003 | 0.001 | -1.29 | 0.197 | 0.592 | 0.276 | -0.093 | [-0.228, 0.005] |
| Change in pre-shot PA (g) | A3 | 167 | 387 | -0.008 | 0.005 | -2.97 | 0.003 | 0.015 | 0.007 | -0.159 | [-0.321, 0.056] |
| Change in pre-shot PA (g) | A4 | 48 | 78 | -0.025 | 0.014 | -4.38 | <0.001 | <0.001 | <0.001 | -0.466 | [-0.626, -0.413] |
| Change in pre-shot PA (g) | A5 | 291 | 584 | 0.000 | 0.002 | -1.16 | 0.246 | 0.592 | 0.288 | -0.048 | [-0.211, 0.096] |
| Change in pre-shot PA (g) | A6 | 196 | 309 | -0.008 | 0.003 | -2.03 | 0.042 | 0.168 | 0.074 | -0.107 | [-0.246, 0.068] |
| Change in pre-shot PA (g) | A7 | 350 | 636 | -0.022 | 0.010 | -6.17 | <0.001 | <0.001 | <0.001 | -0.237 | [-0.327, -0.139] |
| Phasic HR decrease (bpm) | A1 | 109 | 134 | 17.000 | 16.000 | 1.05 | 0.294 | 1.000 | 0.617 | 0.078 | [-0.234, 0.272] |
| Phasic HR decrease (bpm) | A2 | 58 | 85 | 19.000 | 20.000 | -1.31 | 0.190 | 1.000 | 0.617 | -0.129 | [-0.200, -0.022] |
| Phasic HR decrease (bpm) | A3 | 46 | 126 | 17.000 | 14.500 | 0.90 | 0.368 | 1.000 | 0.617 | 0.090 | [-0.029, 1.000] |
| Phasic HR decrease (bpm) | A4 | 33 | 45 | 16.000 | 15.000 | 0.77 | 0.441 | 1.000 | 0.617 | 0.102 | [0.024, 0.400] |
| Phasic HR decrease (bpm) | A5 | 94 | 206 | 17.500 | 16.000 | 1.18 | 0.237 | 1.000 | 0.617 | 0.085 | [-0.180, 0.283] |
| Phasic HR decrease (bpm) | A6 | 166 | 244 | 15.000 | 15.000 | 0.47 | 0.637 | 1.000 | 0.637 | 0.027 | [-0.174, 0.173] |
| Phasic HR decrease (bpm) | A7 | 168 | 347 | 16.000 | 16.000 | -0.49 | 0.626 | 1.000 | 0.637 | -0.026 | [-0.337, 0.082] |
| HR deceleration rate (bpm/s) | A1 | 63 | 95 | 2.143 | 1.833 | 1.20 | 0.231 | 1.000 | 0.525 | 0.113 | [-0.338, 0.307] |
| HR deceleration rate (bpm/s) | A2 | 35 | 37 | 2.222 | 3.143 | -1.65 | 0.100 | 0.699 | 0.525 | -0.225 | [-0.225, -0.181] |
| HR deceleration rate (bpm/s) | A3 | 37 | 92 | 2.000 | 1.944 | -0.07 | 0.944 | 1.000 | 0.944 | -0.008 | [-1.000, -0.000] |
| HR deceleration rate (bpm/s) | A4 | 11 | 17 | 3.000 | 2.714 | 1.04 | 0.300 | 1.000 | 0.525 | 0.235 | [0.042, 0.400] |
| HR deceleration rate (bpm/s) | A5 | 25 | 86 | 2.667 | 2.414 | 0.16 | 0.874 | 1.000 | 0.944 | 0.021 | [-0.259, 0.281] |
| HR deceleration rate (bpm/s) | A6 | 79 | 173 | 2.600 | 2.143 | 1.41 | 0.159 | 0.955 | 0.525 | 0.111 | [-0.258, 0.376] |
| HR deceleration rate (bpm/s) | A7 | 71 | 213 | 3.000 | 2.800 | -0.45 | 0.655 | 1.000 | 0.916 | -0.035 | [-0.146, 0.013] |

**Table S4. Wilcoxon signed-rank tests of the change in pre-shot values against a zero median, separately for putts following a made and following a missed attempt. Here r is the matched-pairs rank-biserial correlation.**

| **Metric** | **Condition** | **Athlete** | **n** | **Median** | **z** | **p** | **p (Holm)** | **p (FDR)** | **r** |
| --- | --- | --- | --- | --- | --- | --- | --- | --- | --- |
| Change in pre-shot HR (bpm) | after a made putt | A1 | 147 | 4.333 | 6.18 | <0.001 | <0.001 | <0.001 | 0.588 |
| Change in pre-shot HR (bpm) | after a made putt | A2 | 101 | 1.833 | 2.28 | 0.023 | 0.045 | 0.026 | 0.261 |
| Change in pre-shot HR (bpm) | after a made putt | A3 | 151 | 0.500 | 1.48 | 0.138 | 0.138 | 0.138 | 0.139 |
| Change in pre-shot HR (bpm) | after a made putt | A4 | 47 | 5.333 | 5.37 | <0.001 | <0.001 | <0.001 | 0.899 |
| Change in pre-shot HR (bpm) | after a made putt | A5 | 221 | 5.333 | 8.76 | <0.001 | <0.001 | <0.001 | 0.679 |
| Change in pre-shot HR (bpm) | after a made putt | A6 | 193 | 1.667 | 3.43 | <0.001 | 0.002 | <0.001 | 0.285 |
| Change in pre-shot HR (bpm) | after a made putt | A7 | 349 | 3.833 | 9.04 | <0.001 | <0.001 | <0.001 | 0.559 |
| Change in pre-shot HR (bpm) | after a missed putt | A1 | 222 | -1.333 | -3.49 | <0.001 | 0.002 | 0.001 | -0.270 |
| Change in pre-shot HR (bpm) | after a missed putt | A2 | 160 | -0.500 | -1.14 | 0.253 | 0.403 | 0.253 | -0.104 |
| Change in pre-shot HR (bpm) | after a missed putt | A3 | 362 | -0.750 | -1.28 | 0.202 | 0.403 | 0.235 | -0.077 |
| Change in pre-shot HR (bpm) | after a missed putt | A4 | 77 | -1.500 | -3.13 | 0.002 | 0.007 | 0.003 | -0.411 |
| Change in pre-shot HR (bpm) | after a missed putt | A5 | 439 | -2.000 | -6.21 | <0.001 | <0.001 | <0.001 | -0.342 |
| Change in pre-shot HR (bpm) | after a missed putt | A6 | 308 | -1.250 | -2.24 | 0.025 | 0.074 | 0.035 | -0.148 |
| Change in pre-shot HR (bpm) | after a missed putt | A7 | 631 | -2.000 | -6.60 | <0.001 | <0.001 | <0.001 | -0.303 |
| Change in pre-shot PA (g) | after a made putt | A1 | 144 | -0.003 | -0.91 | 0.362 | 0.759 | 0.422 | -0.088 |
| Change in pre-shot PA (g) | after a made putt | A2 | 102 | -0.003 | -1.14 | 0.253 | 0.759 | 0.354 | -0.130 |
| Change in pre-shot PA (g) | after a made putt | A3 | 166 | -0.009 | -2.59 | 0.010 | 0.048 | 0.022 | -0.232 |
| Change in pre-shot PA (g) | after a made putt | A4 | 48 | -0.025 | -3.91 | <0.001 | <0.001 | <0.001 | -0.648 |
| Change in pre-shot PA (g) | after a made putt | A5 | 283 | -0.002 | -0.62 | 0.536 | 0.759 | 0.536 | -0.042 |
| Change in pre-shot PA (g) | after a made putt | A6 | 196 | -0.008 | -1.63 | 0.103 | 0.413 | 0.181 | -0.134 |
| Change in pre-shot PA (g) | after a made putt | A7 | 347 | -0.023 | -5.15 | <0.001 | <0.001 | <0.001 | -0.319 |
| Change in pre-shot PA (g) | after a missed putt | A1 | 222 | -0.002 | 0.50 | 0.620 | 1.000 | 0.655 | 0.038 |
| Change in pre-shot PA (g) | after a missed putt | A2 | 168 | 0.002 | 0.45 | 0.655 | 1.000 | 0.655 | 0.040 |
| Change in pre-shot PA (g) | after a missed putt | A3 | 386 | 0.005 | 1.56 | 0.120 | 0.598 | 0.279 | 0.091 |
| Change in pre-shot PA (g) | after a missed putt | A4 | 77 | 0.018 | 2.42 | 0.016 | 0.094 | 0.055 | 0.317 |
| Change in pre-shot PA (g) | after a missed putt | A5 | 578 | 0.002 | 1.16 | 0.248 | 0.831 | 0.347 | 0.055 |
| Change in pre-shot PA (g) | after a missed putt | A6 | 308 | 0.003 | 1.26 | 0.208 | 0.831 | 0.347 | 0.083 |
| Change in pre-shot PA (g) | after a missed putt | A7 | 631 | 0.012 | 3.58 | <0.001 | 0.002 | 0.002 | 0.165 |

# S3. Linear mixed-effects models

All models take the form metric ~ 1 + outcome + (1 | athlete) + (1 | athlete:session) and were fitted by restricted maximum likelihood. Denominator degrees of freedom follow the between-within convention. ICC denotes the proportion of the total variance attributable to the corresponding random effect. The lag-adjusted models additionally include the pre-shot value of the preceding putt as a mean-centred covariate, which absorbs the serial dependence between consecutive attempts.

**Table S5. Fixed-effect estimates from the linear mixed-effects models reported in the manuscript.**

| **Model** | **Term** | **Estimate** | **SE** | **95% CI** | **t** | **df** | **p** | **ICC athlete** | **ICC session** | **n** |
| --- | --- | --- | --- | --- | --- | --- | --- | --- | --- | --- |
| Pre-shot HR ~ outcome of the upcoming putt | outcome | +0.0856 | 0.2807 | [-0.4646, +0.6357] | 0.30 | 3420 | 0.760 | 0.224 | 0.528 | 3451 |
| Pre-shot PA ~ outcome of the upcoming putt | outcome | +0.0036 | 0.0022 | [-0.0008, +0.0079] | 1.60 | 3630 | 0.110 | 0.331 | 0.202 | 3661 |
| Phasic HR decrease ~ outcome of the upcoming putt | outcome | -0.0165 | 0.2981 | [-0.6011, +0.5681] | -0.06 | 1811 | 0.956 | 0.000 | 0.385 | 1842 |
| HR deceleration rate ~ outcome of the upcoming putt | outcome | -0.0128 | 0.0710 | [-0.1522, +0.1266] | -0.18 | 996 | 0.857 | 0.000 | 0.206 | 1027 |
| Pre-shot HR ~ outcome of the upcoming putt, lag-1 adjusted | outcome | +0.0887 | 0.2458 | [-0.3930, +0.5705] | 0.36 | 3374 | 0.718 | 0.168 | 0.371 | 3406 |
| Pre-shot HR ~ outcome of the upcoming putt, lag-1 adjusted | pre-shot value of the preceding putt (centred) | +0.4553 | 0.0153 | [+0.4253, +0.4852] | 29.80 | 3374 | <0.001 | 0.168 | 0.371 | 3406 |
| Pre-shot HR ~ outcome of the preceding putt | outcome | +5.0775 | 0.2754 | [+4.5377, +5.6172] | 18.44 | 3448 | <0.001 | 0.219 | 0.536 | 3479 |
| Pre-shot PA ~ outcome of the preceding putt | outcome | -0.0164 | 0.0022 | [-0.0207, -0.0120] | -7.42 | 3659 | <0.001 | 0.336 | 0.199 | 3690 |
| Change in pre-shot HR ~ outcome of the preceding putt | outcome | +4.8751 | 0.2761 | [+4.3339, +5.4163] | 17.66 | 3402 | <0.001 | 0.000 | 0.000 | 3433 |
| Change in pre-shot PA ~ outcome of the preceding putt | outcome | -0.0196 | 0.0025 | [-0.0244, -0.0148] | -7.95 | 3659 | <0.001 | 0.000 | 0.000 | 3690 |
| Phasic HR decrease ~ outcome of the preceding putt | outcome | +0.2620 | 0.2990 | [-0.3243, +0.8484] | 0.88 | 1830 | 0.381 | 0.000 | 0.392 | 1861 |
| HR deceleration rate ~ outcome of the preceding putt | outcome | +0.0335 | 0.0737 | [-0.1110, +0.1780] | 0.45 | 1003 | 0.649 | 0.000 | 0.204 | 1034 |
| Pre-shot HR ~ outcome of the preceding putt, lag-1 adjusted | outcome | +4.9747 | 0.2376 | [+4.5090, +5.4404] | 20.94 | 3401 | <0.001 | 0.156 | 0.373 | 3433 |
| Pre-shot HR ~ outcome of the preceding putt, lag-1 adjusted | pre-shot value of the preceding putt (centred) | +0.4723 | 0.0147 | [+0.4435, +0.5011] | 32.14 | 3401 | <0.001 | 0.156 | 0.373 | 3433 |
| Pre-shot PA ~ outcome of the preceding putt, lag-1 adjusted | outcome | -0.0177 | 0.0021 | [-0.0217, -0.0137] | -8.62 | 3658 | <0.001 | 0.221 | 0.123 | 3690 |
| Pre-shot PA ~ outcome of the preceding putt, lag-1 adjusted | pre-shot value of the preceding putt (centred) | +0.3724 | 0.0151 | [+0.3427, +0.4020] | 24.59 | 3658 | <0.001 | 0.221 | 0.123 | 3690 |

For reference, the within-session lag-1 autocorrelation of the pre-shot HR across the 29 sessions has a median of 0.40 (interquartile range 0.31 to 0.52; full range 0.01 to 0.77). This is the empirical basis for modelling the clustering explicitly rather than treating putts as independent observations.

# S4. Score-level analyses

The score is the number of consecutive putts already holed when the current attempt is prepared, so a score of 4 means the athlete is attempting the fifth putt that would complete the exercise. Sample sizes decrease sharply with the score by design, and Table S6 reports them for every athlete and score level. Table S7 gives the pairwise post-hoc comparisons following the significant omnibus tests.

**Table S6. Kruskal-Wallis tests of the effect of the running score on the pre-shot metrics, with per-score sample sizes and medians. Benjamini–Hochberg correction is applied across the seven athlete-wise tests within each metric.**

| **Metric** | **Athlete** | **H** | **df** | **p** | **p (FDR)** | **epsilon²** | **n at score 0/1/2/3/4** | **Median at score 0/1/2/3/4** |
| --- | --- | --- | --- | --- | --- | --- | --- | --- |
| Pre-shot HR (bpm) | A1 | 61.2 | 4 | <0.001 | <0.001 | 0.157 | 223 / 88 / 33 / 18 / 7 | 87.33 / 92.92 / 100.67 / 107.33 / 120.50 |
| Pre-shot HR (bpm) | A2 | 17.6 | 4 | 0.001 | 0.002 | 0.052 | 165 / 66 / 24 / 9 / 2 | 94.33 / 96.08 / 99.00 / 103.00 / 144.42 |
| Pre-shot HR (bpm) | A3 | 4.1 | 4 | 0.392 | 0.392 | 0.000 | 374 / 113 / 32 / 9 / 3 | 103.50 / 101.00 / 102.42 / 107.17 / 149.17 |
| Pre-shot HR (bpm) | A4 | 37.7 | 4 | <0.001 | <0.001 | 0.279 | 78 / 31 / 12 / 3 / 2 | 95.42 / 102.00 / 108.08 / 113.00 / 141.92 |
| Pre-shot HR (bpm) | A5 | 100.9 | 4 | <0.001 | <0.001 | 0.141 | 463 / 146 / 54 / 17 / 13 | 105.50 / 112.17 / 115.00 / 128.00 / 133.67 |
| Pre-shot HR (bpm) | A6 | 19.6 | 4 | <0.001 | <0.001 | 0.031 | 309 / 122 / 51 / 17 / 6 | 114.67 / 115.50 / 117.33 / 123.50 / 123.25 |
| Pre-shot HR (bpm) | A7 | 132.4 | 4 | <0.001 | <0.001 | 0.131 | 636 / 214 / 89 / 36 / 12 | 114.50 / 117.67 / 122.17 / 127.25 / 133.83 |
| Change in pre-shot HR (bpm) | A1 | 60.4 | 4 | <0.001 | <0.001 | 0.155 | 223 / 88 / 33 / 18 / 7 | -1.33 / 3.75 / 5.50 / 4.50 / 9.00 |
| Change in pre-shot HR (bpm) | A2 | 11.5 | 4 | 0.022 | 0.022 | 0.029 | 162 / 66 / 24 / 9 / 2 | -0.42 / 1.92 / 1.50 / 2.17 / 42.58 |
| Change in pre-shot HR (bpm) | A3 | 14.2 | 4 | 0.007 | 0.008 | 0.020 | 369 / 110 / 31 / 7 / 3 | -0.33 / -0.25 / 1.83 / 3.00 / 32.00 |
| Change in pre-shot HR (bpm) | A4 | 44.9 | 4 | <0.001 | <0.001 | 0.338 | 78 / 31 / 12 / 3 / 2 | -1.50 / 5.17 / 2.00 / 8.33 / 24.00 |
| Change in pre-shot HR (bpm) | A5 | 124.3 | 4 | <0.001 | <0.001 | 0.183 | 439 / 141 / 52 / 17 / 12 | -2.00 / 5.33 / 5.92 / 4.50 / 5.58 |
| Change in pre-shot HR (bpm) | A6 | 17.9 | 4 | 0.001 | 0.002 | 0.028 | 309 / 122 / 51 / 17 / 6 | -1.17 / 1.25 / 1.00 / 4.00 / 2.00 |
| Change in pre-shot HR (bpm) | A7 | 135.6 | 4 | <0.001 | <0.001 | 0.134 | 636 / 214 / 89 / 36 / 12 | -2.00 / 3.50 / 4.50 / 3.25 / 6.17 |
| Pre-shot PA (g) | A1 | 3.4 | 4 | 0.493 | 0.493 | -0.002 | 223 / 88 / 33 / 18 / 7 | 0.15 / 0.13 / 0.16 / 0.13 / 0.12 |
| Pre-shot PA (g) | A2 | 8.6 | 4 | 0.072 | 0.127 | 0.017 | 170 / 68 / 24 / 9 / 2 | 0.10 / 0.09 / 0.09 / 0.10 / 0.16 |
| Pre-shot PA (g) | A3 | 14.0 | 4 | 0.007 | 0.017 | 0.018 | 387 / 118 / 36 / 9 / 3 | 0.14 / 0.13 / 0.12 / 0.14 / 0.11 |
| Pre-shot PA (g) | A4 | 30.8 | 4 | <0.001 | <0.001 | 0.222 | 78 / 31 / 12 / 3 / 2 | 0.10 / 0.08 / 0.06 / 0.02 / 0.05 |
| Pre-shot PA (g) | A5 | 5.9 | 4 | 0.204 | 0.286 | 0.002 | 584 / 185 / 69 / 23 / 14 | 0.22 / 0.22 / 0.21 / 0.20 / 0.20 |
| Pre-shot PA (g) | A6 | 3.6 | 4 | 0.456 | 0.493 | -0.001 | 309 / 122 / 51 / 17 / 6 | 0.22 / 0.21 / 0.22 / 0.18 / 0.21 |
| Pre-shot PA (g) | A7 | 34.4 | 4 | <0.001 | <0.001 | 0.031 | 636 / 214 / 89 / 36 / 12 | 0.25 / 0.23 / 0.22 / 0.14 / 0.16 |
| Change in pre-shot PA (g) | A1 | 4.2 | 4 | 0.376 | 0.439 | 0.001 | 223 / 88 / 33 / 18 / 7 | -0.00 / -0.00 / 0.00 / -0.02 / -0.00 |
| Change in pre-shot PA (g) | A2 | 8.4 | 4 | 0.078 | 0.137 | 0.016 | 170 / 68 / 24 / 9 / 2 | 0.00 / -0.01 / 0.01 / 0.02 / 0.03 |
| Change in pre-shot PA (g) | A3 | 12.2 | 4 | 0.016 | 0.037 | 0.015 | 387 / 118 / 36 / 9 / 3 | 0.00 / -0.01 / -0.01 / -0.01 / -0.05 |
| Change in pre-shot PA (g) | A4 | 26.7 | 4 | <0.001 | <0.001 | 0.188 | 78 / 31 / 12 / 3 / 2 | 0.01 / -0.03 / -0.00 / -0.03 / 0.03 |
| Change in pre-shot PA (g) | A5 | 2.8 | 4 | 0.587 | 0.587 | -0.001 | 584 / 185 / 69 / 23 / 14 | 0.00 / 0.00 / -0.00 / -0.00 / 0.00 |
| Change in pre-shot PA (g) | A6 | 4.5 | 4 | 0.343 | 0.439 | 0.001 | 309 / 122 / 51 / 17 / 6 | 0.00 / -0.01 / 0.00 / -0.02 / 0.00 |
| Change in pre-shot PA (g) | A7 | 46.5 | 4 | <0.001 | <0.001 | 0.043 | 636 / 214 / 89 / 36 / 12 | 0.01 / -0.03 / -0.00 / -0.02 / -0.05 |

**Table S7. Dunn post-hoc comparisons of the pre-shot HR between score levels, within each athlete. Holm correction is applied across the pairwise comparisons within each athlete. Comparisons involving fewer than three observations in either group are omitted.**

| **Athlete** | **Score comparison** | **n₁** | **n₂** | **z** | **p** | **p (Holm)** |
| --- | --- | --- | --- | --- | --- | --- |
| A1 | 0 vs 1 | 223 | 88 | -2.13 | 0.033 | 0.132 |
| A1 | 0 vs 2 | 223 | 33 | -5.15 | <0.001 | <0.001 |
| A1 | 0 vs 3 | 223 | 18 | -5.28 | <0.001 | <0.001 |
| A1 | 0 vs 4 | 223 | 7 | -3.91 | <0.001 | <0.001 |
| A1 | 1 vs 2 | 88 | 33 | -3.39 | <0.001 | 0.004 |
| A1 | 1 vs 3 | 88 | 18 | -3.96 | <0.001 | <0.001 |
| A1 | 1 vs 4 | 88 | 7 | -3.14 | 0.002 | 0.008 |
| A1 | 2 vs 3 | 33 | 18 | -1.13 | 0.256 | 0.581 |
| A1 | 2 vs 4 | 33 | 7 | -1.30 | 0.194 | 0.581 |
| A1 | 3 vs 4 | 18 | 7 | -0.47 | 0.640 | 0.640 |
| A2 | 0 vs 1 | 165 | 66 | -1.83 | 0.068 | 0.250 |
| A2 | 0 vs 2 | 165 | 24 | -1.96 | 0.050 | 0.248 |
| A2 | 0 vs 3 | 165 | 9 | -2.71 | 0.007 | 0.040 |
| A2 | 1 vs 2 | 66 | 24 | -0.68 | 0.495 | 0.495 |
| A2 | 1 vs 3 | 66 | 9 | -1.86 | 0.062 | 0.250 |
| A2 | 2 vs 3 | 24 | 9 | -1.28 | 0.201 | 0.403 |
| A3 | 0 vs 1 | 374 | 113 | 1.43 | 0.153 | 1.000 |
| A3 | 0 vs 2 | 374 | 32 | -0.28 | 0.780 | 1.000 |
| A3 | 0 vs 3 | 374 | 9 | 0.28 | 0.781 | 1.000 |
| A3 | 0 vs 4 | 374 | 3 | -1.29 | 0.198 | 1.000 |
| A3 | 1 vs 2 | 113 | 32 | -1.02 | 0.306 | 1.000 |
| A3 | 1 vs 3 | 113 | 9 | -0.17 | 0.864 | 1.000 |
| A3 | 1 vs 4 | 113 | 3 | -1.54 | 0.124 | 1.000 |
| A3 | 2 vs 3 | 32 | 9 | 0.39 | 0.700 | 1.000 |
| A3 | 2 vs 4 | 32 | 3 | -1.15 | 0.250 | 1.000 |
| A3 | 3 vs 4 | 9 | 3 | -1.26 | 0.208 | 1.000 |
| A4 | 0 vs 1 | 78 | 31 | -3.52 | <0.001 | 0.002 |
| A4 | 0 vs 2 | 78 | 12 | -4.26 | <0.001 | <0.001 |
| A4 | 0 vs 3 | 78 | 3 | -3.22 | 0.001 | 0.005 |
| A4 | 1 vs 2 | 31 | 12 | -1.69 | 0.092 | 0.184 |
| A4 | 1 vs 3 | 31 | 3 | -1.90 | 0.058 | 0.173 |
| A4 | 2 vs 3 | 12 | 3 | -0.89 | 0.373 | 0.373 |
| A5 | 0 vs 1 | 463 | 146 | -5.54 | <0.001 | <0.001 |
| A5 | 0 vs 2 | 463 | 54 | -5.75 | <0.001 | <0.001 |
| A5 | 0 vs 3 | 463 | 17 | -5.70 | <0.001 | <0.001 |
| A5 | 0 vs 4 | 463 | 13 | -5.32 | <0.001 | <0.001 |
| A5 | 1 vs 2 | 146 | 54 | -1.89 | 0.058 | 0.121 |
| A5 | 1 vs 3 | 146 | 17 | -3.44 | <0.001 | 0.004 |
| A5 | 1 vs 4 | 146 | 13 | -3.35 | <0.001 | 0.004 |
| A5 | 2 vs 3 | 54 | 17 | -2.08 | 0.037 | 0.121 |
| A5 | 2 vs 4 | 54 | 13 | -2.17 | 0.030 | 0.121 |
| A5 | 3 vs 4 | 17 | 13 | -0.24 | 0.808 | 0.808 |
| A6 | 0 vs 1 | 309 | 122 | -1.56 | 0.120 | 0.478 |
| A6 | 0 vs 2 | 309 | 51 | -1.98 | 0.047 | 0.237 |
| A6 | 0 vs 3 | 309 | 17 | -2.89 | 0.004 | 0.035 |
| A6 | 0 vs 4 | 309 | 6 | -2.95 | 0.003 | 0.032 |
| A6 | 1 vs 2 | 122 | 51 | -0.80 | 0.425 | 0.594 |
| A6 | 1 vs 3 | 122 | 17 | -2.14 | 0.033 | 0.229 |
| A6 | 1 vs 4 | 122 | 6 | -2.51 | 0.012 | 0.097 |
| A6 | 2 vs 3 | 51 | 17 | -1.50 | 0.134 | 0.478 |
| A6 | 2 vs 4 | 51 | 6 | -2.12 | 0.034 | 0.229 |
| A6 | 3 vs 4 | 17 | 6 | -1.04 | 0.297 | 0.594 |
| A7 | 0 vs 1 | 636 | 214 | -4.70 | <0.001 | <0.001 |
| A7 | 0 vs 2 | 636 | 89 | -8.03 | <0.001 | <0.001 |
| A7 | 0 vs 3 | 636 | 36 | -6.85 | <0.001 | <0.001 |
| A7 | 0 vs 4 | 636 | 12 | -5.38 | <0.001 | <0.001 |
| A7 | 1 vs 2 | 214 | 89 | -4.26 | <0.001 | <0.001 |
| A7 | 1 vs 3 | 214 | 36 | -4.46 | <0.001 | <0.001 |
| A7 | 1 vs 4 | 214 | 12 | -4.03 | <0.001 | <0.001 |
| A7 | 2 vs 3 | 89 | 36 | -1.34 | 0.180 | 0.359 |
| A7 | 2 vs 4 | 89 | 12 | -2.14 | 0.032 | 0.097 |
| A7 | 3 vs 4 | 36 | 12 | -1.18 | 0.238 | 0.359 |

# S5. Session-level results

The table below gives, for each of the 29 sessions, the change in pre-shot HR after a made and after a missed putt, tested against a zero median. The direction of the effect is consistent in 26 of the 29 sessions in both conditions, whereas statistical significance is reached in fewer, largely as a function of session length.

**Table S8. Session-level Wilcoxon signed-rank tests of the change in pre-shot HR against a zero median. Benjamini–Hochberg correction is applied across the 29 sessions within each condition.**

| **Session** | **n after made** | **Median (bpm)** | **p** | **p (FDR)** | **n after missed** | **Median (bpm)** | **p** | **p (FDR)** |
| --- | --- | --- | --- | --- | --- | --- | --- | --- |
| A1-S1 | 22 | 2.25 | 0.085 | 0.121 | 16 | -1.25 | 0.408 | 0.514 |
| A1-S2 | 41 | 2.17 | 0.028 | 0.047 | 69 | -0.17 | 0.404 | 0.514 |
| A1-S3 | 67 | 7.20 | <0.001 | <0.001 | 107 | -1.67 | 0.001 | 0.011 |
| A1-S4 | 13 | 4.00 | 0.081 | 0.121 | 13 | -1.33 | 0.529 | 0.614 |
| A1-S5 | 4 | – | – | – | 17 | -2.50 | 0.149 | 0.254 |
| A2-S1 | 59 | 0.83 | 0.797 | 0.828 | 74 | 0.17 | 0.916 | 0.916 |
| A2-S2 | 42 | 3.42 | <0.001 | <0.001 | 86 | -1.17 | 0.069 | 0.143 |
| A3-S1 | 113 | 0.17 | 0.740 | 0.799 | 257 | -0.67 | 0.363 | 0.501 |
| A3-S2 | 27 | 2.00 | 0.033 | 0.053 | 58 | -1.75 | 0.109 | 0.211 |
| A3-S3 | 11 | 3.67 | 0.374 | 0.481 | 47 | 2.67 | 0.703 | 0.755 |
| A4-S1 | 28 | 5.25 | <0.001 | <0.001 | 49 | -1.17 | 0.026 | 0.067 |
| A4-S2 | 14 | 4.67 | <0.001 | 0.002 | 13 | -4.17 | 0.046 | 0.108 |
| A4-S3 | 5 | – | – | – | 15 | -1.50 | 0.256 | 0.391 |
| A5-S1 | 14 | 6.33 | 0.002 | 0.004 | 30 | -0.92 | 0.144 | 0.254 |
| A5-S2 | 30 | 7.83 | <0.001 | <0.001 | 51 | -3.33 | 0.006 | 0.025 |
| A5-S3 | 89 | 6.17 | <0.001 | <0.001 | 166 | -2.42 | <0.001 | <0.001 |
| A5-S4 | 37 | 4.00 | 0.015 | 0.026 | 106 | -1.58 | 0.023 | 0.066 |
| A5-S5 | 19 | 1.33 | 0.147 | 0.199 | 21 | -0.83 | 0.244 | 0.391 |
| A5-S6 | 32 | 3.33 | 0.008 | 0.015 | 65 | -2.00 | 0.048 | 0.108 |
| A6-S1 | 70 | 2.75 | 0.002 | 0.004 | 127 | -1.83 | 0.020 | 0.063 |
| A6-S2 | 54 | -0.08 | 0.721 | 0.799 | 93 | 0.50 | 0.333 | 0.483 |
| A6-S3 | 24 | 6.83 | <0.001 | 0.002 | 20 | -3.25 | 0.005 | 0.023 |
| A6-S4 | 13 | 0.50 | 0.576 | 0.676 | 9 | -2.17 | 0.594 | 0.662 |
| A6-S5 | 32 | 0.67 | 0.955 | 0.955 | 59 | -0.67 | 0.771 | 0.799 |
| A7-S1 | 92 | 3.50 | <0.001 | <0.001 | 171 | -1.33 | 0.004 | 0.023 |
| A7-S2 | 56 | 2.75 | <0.001 | <0.001 | 82 | -2.17 | 0.007 | 0.025 |
| A7-S3 | 88 | 4.25 | <0.001 | <0.001 | 118 | -2.00 | 0.001 | 0.011 |
| A7-S4 | 24 | 1.75 | 0.530 | 0.650 | 44 | -1.67 | 0.434 | 0.525 |
| A7-S5 | 89 | 5.50 | <0.001 | <0.001 | 216 | -2.50 | <0.001 | <0.001 |
